# Disentangling Direct from Indirect Co-Evolution of Residues in Protein Alignments

**DOI:** 10.1371/journal.pcbi.1000633

**Published:** 2010-01-01

**Authors:** Lukas Burger, Erik van Nimwegen

**Affiliations:** Biozentrum, University of Basel, and Swiss Institute of Bioinformatics, Basel, Switzerland; University of California San Diego, United States of America

## Abstract

Predicting protein structure from primary sequence is one of the ultimate challenges in computational biology. Given the large amount of available sequence data, the analysis of co-evolution, i.e., statistical dependency, between columns in multiple alignments of protein domain sequences remains one of the most promising avenues for predicting residues that are contacting in the structure. A key impediment to this approach is that strong statistical dependencies are also observed for many residue pairs that are distal in the structure. Using a comprehensive analysis of protein domains with available three-dimensional structures we show that co-evolving contacts very commonly form chains that percolate through the protein structure, inducing indirect statistical dependencies between many distal pairs of residues. We characterize the distributions of length and spatial distance traveled by these co-evolving contact chains and show that they explain a large fraction of observed statistical dependencies between structurally distal pairs. We adapt a recently developed Bayesian network model into a rigorous procedure for disentangling direct from indirect statistical dependencies, and we demonstrate that this method not only successfully accomplishes this task, but also allows contacts with weak statistical dependency to be detected. To illustrate how additional information can be incorporated into our method, we incorporate a phylogenetic correction, and we develop an informative prior that takes into account that the probability for a pair of residues to contact depends strongly on their primary-sequence distance and the amount of conservation that the corresponding columns in the multiple alignment exhibit. We show that our model including these extensions dramatically improves the accuracy of contact prediction from multiple sequence alignments.

## Author Summary

Whenever two residues are in close contact in the structure of a protein, their interaction will often constrain which amino acid substitutions can occur without perturbing the functionality of the protein, leading to “co-evolution” of the residues. With the large amount of data currently available, deep multiple alignments can be constructed of protein sequences that likely fold into a common structure, and several methods have been proposed for predicting contacting residues from statistical dependencies exhibited by pairs of alignment columns. Unfortunately, strong statistical dependencies are also observed between many pairs of residues that are distal in the structure. Through a comprehensive analysis of 2009 protein domains, we show that a large fraction of these distal dependencies are indirect and result from chains of contacting pairs that percolate through the protein. We present a Bayesian network model that rigorously disentangles direct from indirect dependencies and show that this greatly improves contact prediction. Additionally, we develop an informative prior that takes into account that the probability for residues to be in contact depends on their primary sequence separation, and that highly conserved residues tend to participate in a larger number of contacts. With this prior, the accuracy of the contact predictions is dramatically improved.

## Introduction

The identification of functionally and structurally important elements in DNA, RNA and proteins from their sequences has been a major focus of computational biology for several decades. A common approach is to create a multiple alignment of homologous sequences, which places ‘equivalent’ residues into the same column and as such gives a hint of the evolutionary constraints that are acting on related sequences. In particular, so-called profile hidden Markov models [1] of protein families and domains have been highly successful in identifying sequences that have similar function and fold into a common structure, making them among the most important tools in functional genomics, see e.g. [2]. These hidden Markov models typically assume that the residues occurring at a given position are probabilistically independent of the residues occurring at other positions. At the time at which these models were developed, it was entirely reasonable to ignore dependencies between residues at different positions, since the amount of available sequence data was generally insufficient to estimate joint probabilities of multiple residues. However, currently the multiple alignments of many protein families and domains include hundreds and sometimes even thousands of sequences, making it possible to systematically investigate dependencies between the residues at different positions.

As the functionality of biomolecules crucially depends on their three-dimensional structures, whose stabilities depend on interactions between residues that are near to each other in space, it is of course to be expected that significant dependencies between residues at different positions will exist. Indeed such dependencies are evident for RNA (eg [3],[4]) and protein sequences [5],[6]. The existence of dependencies between residues at different positions is also supported by the observation of correlated mutations in which mutations at one residue tend to be compensated by a correlated mutation in a particular other residue [5]–[7].

**Figure 1:**
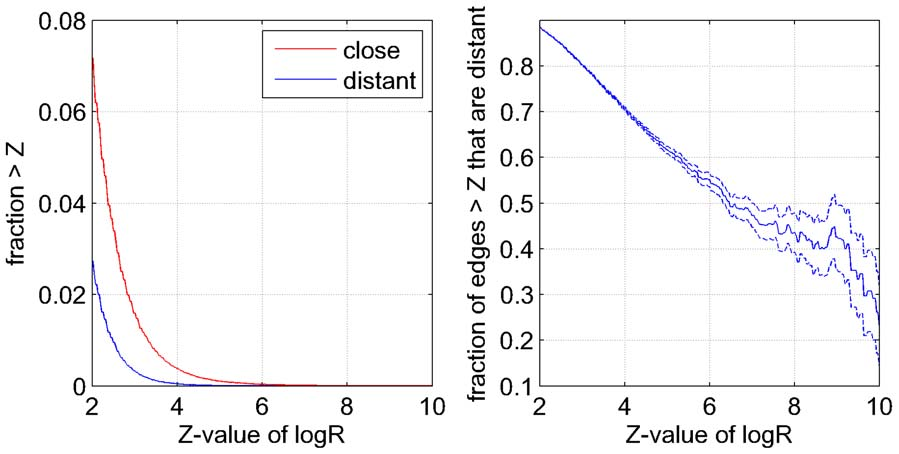
Statistical dependencies of structurally close and distal residue pairs. Left panel: Reverse-cumulative distribution of 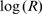 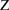-values (horizontal axis) for structurally close (red) and distal (blue) residue pairs. Right panel: The fraction of all residue pairs that are distal in the structure as a function of their statistical dependency (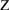-value).

Recently there has been a significant amount of work in which multiple alignments of single protein families have been used in order to predict pairs of residues that are functionally linked or interact directly in the tertiary structure (see eg [8]–[14] and references therein). This work has shown that pairs of residues which show statistical dependencies are generally significantly closer in the structure than randomly chosen pairs. However, it has been repeatedly noted that there exist many highly statistically-dependent residues that are distant in space (eg [14]–[16]). Figure 1 illustrates these points. One of the most commonly used measures of dependency between two residues is the mutual information [4],[9],[14],[17],[18] between the distributions of amino acids occurring in the two corresponding alignment columns. We collected a comprehensive set of 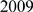 multiple alignments of protein domains from the Pfam database [19] for which a three dimensional structure was available (see *Materials and Methods*) and calculated, for each pair 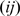 of columns in each alignment, the statistical dependency using a measure, 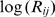, which is a finite-size corrected version of mutual information (see *Materials and Methods*). Since the distribution of 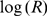 values for an alignment depends strongly on the number of sequences in the alignment, their phylogenetic relationship, and the length of the alignment, 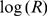 values cannot be directly compared across different alignments. Therefore, we calculated the mean and variance of 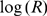 values for each alignment and transformed the 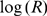 values to 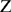-values (number of standard deviations from the mean). Finally, for each alignment, we divided all pairs of residues into those that are contacting in the three-dimensional structure, and those that are distant in the structure, and calculated the distribution of 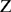-values for these two sets of residue pairs. As in previous work (e.g. [10],[20]) and as defined for CASP [21], two residues were considered in contact if their 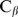 distance (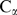 for glycines) in the structure was smaller than 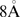. Combining the data from all alignments, the left panel of Figure 1 shows the fraction of all pairs of contacting residues (red) and distal residues (blue) larger than a given 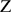-value as a function of 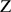. The right panel shows, as a function of 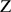, what fraction of all residue pairs with at least this 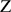-value are contacting in the structure.

The left panel of Figure 1 illustrates that, indeed, a higher fraction of contacting residues shows strong statistical dependencies than distal residues. However, we also see that the difference in the 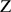-distribution of close and distal pairs is only moderate. Since there are generally many more distal pairs than close pairs, this implies that, even at high 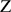-values, the majority of residue-pairs are in fact distal in the structure (Figure 1, right panel). This result shows that simple measures of statistical dependency, such as mutual information, are poor at predicting which pairs of residues are directly contacting in the structure.

The main question is why so many structurally distal pairs show statistical dependencies in their amino-acid distributions that are stronger than those between directly contacting residues. First, whereas measures such as mutual information treat the sequences in the multiple alignments as statistically independent, in reality many of the sequences are phylogenetically closely related, which can cause ‘spurious’ statistical dependencies to appear between independent residue pairs which can be larger than the true statistical dependencies between contacting pairs. Several groups have investigated this confounding factor in contact prediction and several methods have been proposed for correcting these spurious phylogenetic correlations [8],[9],[13],[14], which we will make use of below.

Although important, many strong statistical dependencies between distal residues remain even when spurious phylogenetic dependencies are corrected for (see below). Some of these distant dependencies have been suggested to be caused by homo-oligomeric interactions [14],[22]. Thus, in this interpretation, some of the ‘distal’ pairs with strong statistical dependencies are in fact contacting in the homo-oligomer. Although it is not clear how many of the distal dependencies can be explained by this mechanism, it seems likely that only a relatively small number of residue pairs on the surface can be responsible for such homo-oligomeric interactions.

A third explanation that has been offered for the large number of distal pairs with strong statistical dependencies is that these dependencies are induced by *indirect* interactions that are mediated either by intermediate molecules [15],[23] or by chains of directly interacting residue pairs that run through the protein and connect distal pairs [23]–[25]. Indeed, for a small number of example domains, the existence of such chains of thermodynamically directly coupled residues has been demonstrated [23],[24]. However, the connection between thermodynamic coupling and covariation is still under debate as there is little evidence that thermodynamic coupling of residues is limited to covarying positions [26].

**Figure 2:**
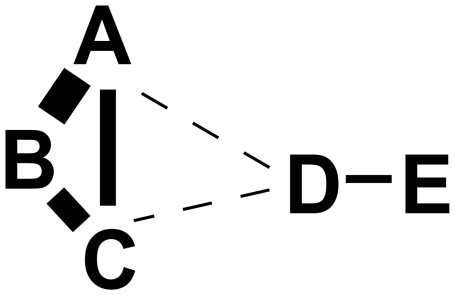
Statistical dependencies between pairs of residues reflect both direct and indirect interactions. The 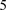 letters (A through E) represent 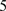 residues and their distances in the figure reflect their distances in the three-dimensional structure. We assume that the pairs A–B, B–C, and D–E are in contact and interact directly. The thickness of the edges between pairs of nodes reflect the statistical dependencies between the corresponding columns in the multiple alignment.

In this paper, we comprehensively investigate to what extent statistical dependencies between distal pairs can be explained by indirect dependencies. The conceptual idea is illustrated in figure 2.

In this illustration, the letters reflect different residues, their distances in the figure reflect their distances in the three dimensional structure, i.e. only the pairs A–B, B–C, and D–E interact directly, and the strength of the statistical dependencies between the different pairs are represented by the thickness of the lines connecting them. Because the pairs A–B and B–C have very high statistical dependency, a strong dependency between A and C is *induced*, which is larger even than the statistical dependency of the directly interacting pair D–E. Any method that considers the statistical dependencies of each pair independently would thus erroneously assign higher confidence to the interaction of A–C than that of D–E.

It should be noted that mutual information and variants thereof have been used extensively for the inference of interacting nucleic acid pairs (see [4] for a review) in the secondary structures of RNA sequences. In these approaches too, the significance of the statistical dependency between a pair of potentially interacting positions is typically evaluated in isolation, i.e. independent of the dependencies between all other pairs. However, in contrast to protein structures, RNA secondary structures per definition consist of *disjoint pairs* of directly interacting residues, i.e. those that form Watson-Crick base pairs. Thus, for RNA secondary structures the ‘percolation’ of statistical dependencies to pairs that are distal in the structure cannot occur (ignoring tertiary structure).

Below we show that chains of statistically dependent contacts are very common in protein structures, explaining a significant fraction of observed dependencies between structurally distal pairs, and we characterize the distribution of lengths and distance traveled by such chains. We show that a Bayesian network model which we recently developed to predict protein-protein interactions [27] can be adapted to rigorously disentangle direct from indirect statistical dependencies between residues, and we demonstrate that such an approach much improves the prediction of pairs of residues that are in contact in the three-dimensional structure. We then investigate to what extent our Bayesian network algorithm can be further improved by incorporating a correction for the phylogenetic dependencies between sequences in the alignment [14], and by incorporating prior information regarding possible interactions. In particular we develop an informative prior that incorporates the observations that the probability for two residues to interact depends strongly on their distance in the primary sequence, and that highly conserved positions in the multiple alignment tend to interact with a higher number of other residues. We show that incorporating these additional features into our Bayesian network model dramatically improves the accuracy of the predictions.

## Results

### Distant co-evolving pairs can frequently be explained by chains of co-evolving contacts

**Figure 3:**
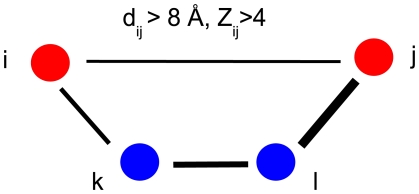
Illustration of a chain that explains the dependency between two distant residues 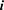 and 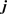. The distance between the nodes illustrates the spatial separation and the thickness of the edges represents the strength of the dependence. Nodes 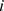 and 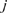 can be connected indirectly via a chain of contacts (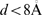) through nodes 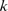 and 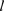 (in blue) whose edges all have higher dependency (i.e. 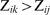, 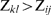 and 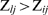).

As mentioned above, it has been suggested that statistical dependencies between structurally distant residue pairs can be explained by chains of contacts that are all statistically dependent. However, the existence of such ‘co-evolving chains’ of contacts has only been demonstrated for a small number of examples [23],[24]. To examine comprehensively and systematically to what extent statistical dependencies between structurally distal residues can be explained by co-evolving chains of contacts we extracted, for each multiple alignment, all pairs of residues that showed high statistical dependency (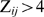). We then divided these ‘co-evolving pairs’ into co-evolving contacts and co-evolving distal pairs. As illustrated in Figure 3, we then determined for each distal pair whether there exists a chain of contacts that each show stronger co-evolution than the distal pair, i.e. 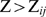 for all contacts in the chain.

However, since our 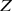-values are in all likelihood only a very noisy measure of the true co-evolution of pairs, we expect that frequently one or more of the contacts in the chain may have a lower 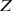-value, even if their true co-evolution is higher than the co-evolution of pair 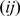. We therefore also consider chains where some contacts 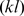 have 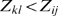 and define the total score 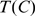 of a chain 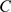 as the sum of the difference in 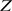-value for all edges that have lower 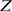-value than the distal pair 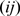, i.e
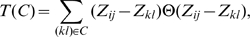
(1)where 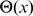 is the Heaviside-function which is one when 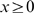 and zero otherwise. For each distal co-evolving pair, we determined the chain of contacts 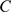 that has minimal total score 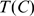. Since pairs that are very distal per definition require longer chains, and since 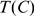 generally grows with the length of the chain, we define the final score 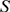 of the best path for a given pair as the average score per contact, i.e. 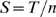, where 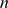 is the number of contacts in the best path.

**Figure 4:**
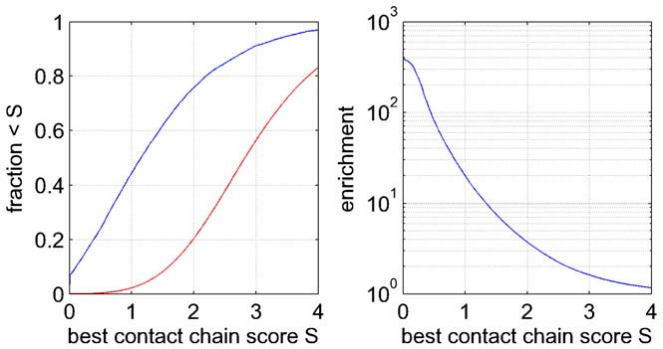
Most distal co-evolving pairs can be explained by chains of co-evolving contacts. Left panel: Cumulative distributions for the number of distal pairs 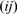 (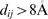) that co-evolve (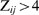) that can be explained by chains of co-evolving contacts as a function of the score 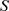 of the best chain (see text). The blue line shows the distribution for the true data and the red curve for the randomized data. Right panel: Ratio (fold-enrichment) of the fraction of distal co-evolving pairs that can be explained by chains versus the fraction that can be explained by chains from the randomized data. The vertical axis is shown on a logarithmic scale.

The left panel of Figure 4 shows the cumulative distribution of the scores 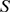 of the best chains (blue curve). We see that for 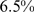 of the distal co-evolving pairs, there exists a chain with score 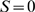, i.e. where all contacts in the chain have 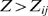. The median score of the best contact path is a little larger than 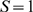, and the 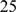
th and 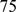 percentiles occur at 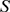-values of about 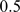 and 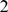 respectively. Note that, as all distal co-evolving pairs have 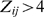, even at a score of 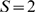 the contacts in the path have 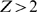 on average, meaning that they are still among the most significantly co-evolving pairs.

To assess the significance of the cumulative distribution 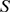 we performed a randomization test by randomly permuting the 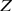-values of all contacts of each domain 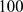 times and determining the 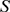 scores of the best paths that are obtained with these permuted 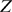-values. The red curve in the left panel of Figure 4 shows the cumulative distribution of 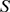-scores obtained in this randomized set and it is immediately clear that the 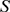-scores are much higher for the randomized set. The right panel of Figure 4 shows, as a value of 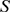, the ratio between the fraction of distal pairs that can be explained by a chain with score less than 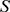 for the real and the randomized data. Especially at low values of 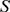 the ratios are enormous. For example, at 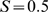 the ratio is about 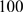, meaning that whereas about 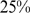 of the distal pairs can be explained by chains in the real data, in the randomized data virtually no distal pairs can be explained, i.e. only 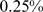. But strong enrichment persists until much higher values of 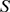. For example, at 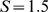 about two-thirds of distal pairs can be connected by a chain, whereas the percentage is less than 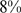 for the randomized data.

### Statistics of co-evolving contact chains

**Figure 5:**
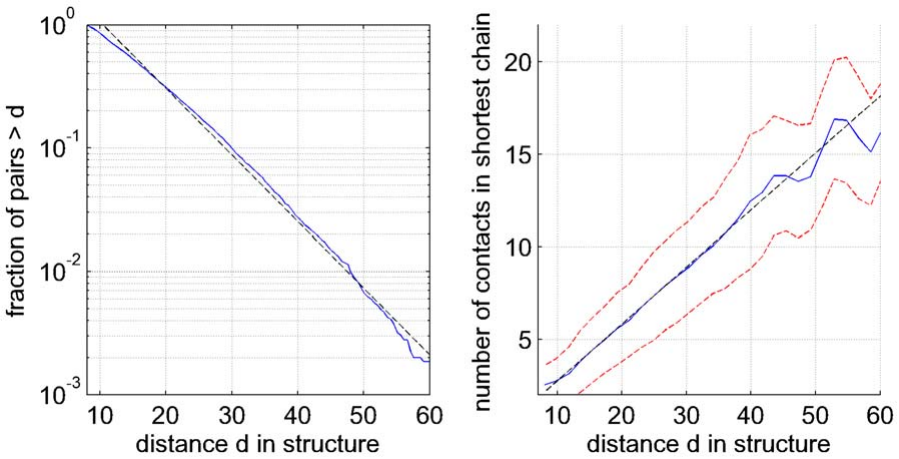
Statistics of co-evolving contact chains. Left panel: Reverse-cumulative distribution of the spatial distances between co-evolving pairs that can be explained by chains of co-evolving contacts of score 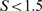. The vertical axis is shown on a logarithmic scale. The dotted line shows a fit to an exponential distribution 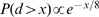. Right panel: Number of steps in the shortest co-evolving contact chain as a function of the spatial distance of the co-evolving pair. The blue line shows the mean distance and the red dotted lines show mean plus and minus one standard deviation. The black dotted line shows a linear fit, the fitted slope of which corresponds to an increase in distance by 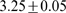
Å per additional contact in the chain.

Our results show that, across essentially all protein domains for which multiple alignments and structures are available, chains of co-evolving contacts are common and explain a large fraction of statistical dependencies observed between structurally distal pairs. To gain insights in the nature of these co-evolving contact chains in protein structures, we selected all distal pairs that are explained by contact chains with scores 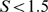 and obtained statistics on the number of steps and the spatial distance covered by these chains (Figure 5).

We see that the distance distribution of ‘explainable’ distal co-evolving pairs is roughly exponential with a length scale of about 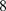 Å. Since ‘distal pairs’ are by definition at least 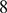
Å apart, this means that the typical length scale covered by co-evolving contact chains is about 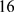
Å. The right panel of Figure 5 shows the mean number of steps in the shortest co-evolving contact chain as a function of the structural distance of the co-evolving distal pair. With increasing spatial separation, the number of edges in the chain steadily increases from on average 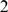 steps at a separation of 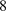
Å to 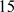 steps at 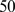
Å. Interestingly, the increase in the average number of steps as a function of distance is almost perfectly linear and corresponds to 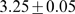
Å per step. We thus see that ‘typical’ co-evolving contact paths contain about 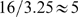 steps, demonstrating that statistical dependencies typically percolate along paths with multiple steps. We also note that some chains are very long, consisting of up to 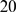 steps, connecting residues that are as far as 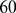
Å apart in the structure.

### Bayesian network model

The insight that many of the statistical dependencies between structurally distal pairs result from chains of co-evolving contacts has important consequences for contact prediction methods. That is, any method that aims to predict contacting residues from statistical dependencies should clearly take into account indirect dependencies that are induced by such chains.

In [27] we developed a general Bayesian network model for calculating the probability of a multiple alignment of protein sequences taking into account dependencies between amino acids at all possible pairs of positions. We refer the reader to [27] for a comprehensive explanation of the method. Briefly, our model assumes that the sequences in a multiple alignment 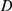 (the data) are drawn from an (unknown) underlying joint probability distribution 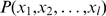 with 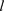 the width of the alignment and 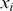 the amino acid at position 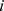. Profile hidden Markov models typically assume that the amino acids at different positions are independent so that one can write 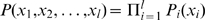, with 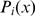 the probability distribution of amino acids at position 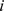. Note that, since there are 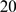 amino acids (disregarding gaps), such models will have 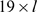 parameters in total. Our model of 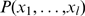 allows general dependencies, such that the probability for an amino acid at position 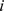 depends on the amino acids at other positions. Note that, if the residue at 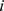 is dependent on a residue at one single other position 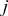, there are already 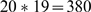 parameters in the distribution 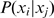, and that models with dependencies on two other positions, i.e. 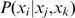, would have 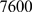 parameters for each residue. Given the current amount of sequence data, it is certainly reasonable to consider models with single dependencies, but there is hardly ever enough data to meaningfully estimate 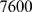 parameters per position. Our model therefore only considers pairwise conditional dependencies of the form 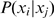.

Any model that considers only pairwise conditional dependencies factorizes the joint probability 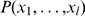 as a product 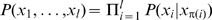, where 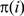 is the single other position which the residue at position 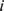 depends on (note that independence, i.e. 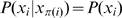 is contained in this general model). Our Bayesian network model is the most general model of this form. In particular, we do not attempt to estimate the conditional probabilities 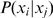 but rather treat these conditional probabilities as nuisance parameters that we integrate out in calculating the likelihood of the alignment. In addition, and importantly, we do not consider only a single ‘best’ way of choosing which other position 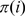 each position 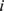 depends on, but rather we *sum* over all ways in which the dependencies can be chosen. Note that if we consider each column of the alignment as a node in a graph and connect each node 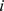 to the node it depends on, 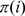, then any consistent set of dependencies 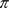, i.e. any set of dependencies 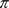 that does not introduce cycles in the graph, corresponds to a *spanning tree* of this graph. Thus, the sum over all consistent ways in which we can assign dependencies is in fact the sum over the set of all possible spanning trees of our graph. As explained in [27] and the *Materials and Methods* section, all integrals over the unknown conditional probabilities 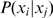 can be performed analytically and, importantly, the sum over all spanning trees can be calculated as a matrix determinant using a generalization of Kirchhoff's theorem [28]. It is thus feasible to do inference with this general Bayesian network for a large number of multiple alignments, including alignments that are hundreds of columns wide.

### Posterior probability of a pairwise interaction

**Figure 6:**
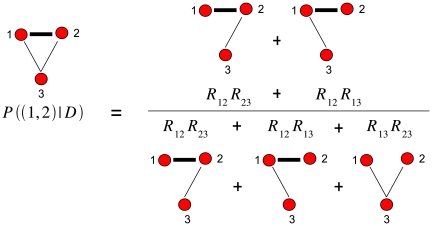
Illustration of the calculation of the posterior probability. For the sake of simplicity, we here show an example for an alignment with only 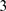 columns. The posterior probability for edge 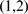 is the statistical weight of all spanning trees that contain this edge relative to the weight of all possible spanning trees.

In our model the joint probability of a multiple alignment is given as the sum over all possible spanning trees of node-dependencies, where each spanning tree is weighted according to the product of statistical dependencies across all edges in the tree (see *Materials and Methods*). Here the statistical dependence between any pair of positions 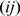 is given by the ratio 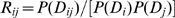 of the joint probability of the alignment columns 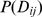 and the product 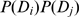 of their marginal probabilities. Since the number of edges in any spanning tree is limited, there is a natural ‘competition’ in this model between the edges to be included in the spanning tree. Therefore, spanning trees with the highest statistical weight will only use edges whose statistical dependence can *not* be explained by chains of other edges with higher dependency, and edges between pairs with indirect statistical dependency will thus only appear in spanning trees with relatively low statistical weight. The posterior probability 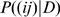, given the data 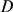, for a pair 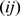 to interact directly can thus very naturally be quantified within our model by calculating the sum of the statistical weights of all spanning trees in which the edge between the pair 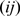 exists. The calculation of this posterior is illustrated in Figure 6.

Note that in this calculation 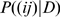 depends on the statistical dependencies between all pairs of positions and that all possible spanning trees are included in the calculation. Roughly speaking, a high posterior 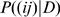 indicates that the edge 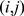 is included in most spanning trees that have high probability. In this way indirect dependencies are accounted for in a rigorous way, derived from first principles, and without any free parameters.

### Posterior probabilities significantly improve contact predictions

To compare the performance of the traditional mutual information-based measurement with the predictions of our model, we calculated mutual information 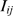, our analogous measure 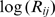, as well as the posterior probabilities 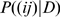 for each pair of positions 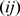 for each domain in our set of 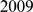 Pfam alignments with available three dimensional structure.

Different domains have widely varying widths and also widely varying numbers of sequences in the alignments. With regard to the former, it is well-known that the number of pairs that are in contact in three-dimensional protein structures increases with the length of the protein sequence. To compare prediction accuracies for proteins with different lengths, the consensus, also used by the CASP assessors [21], has been to compare the number of predictions per residue. However, although there is a large variation across domains, we find that the number of contacts scales slightly super-linearly, with an exponent of roughly 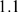 for all pairs of residues, and up to 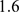 if we consider only pairs of residues that are distal in the primary sequence (see Figure S1). That is, the number of contacts per residue grows with the length of the domain, making it problematic to use predictions-per-residue as a common reference for domains of different length. We therefore decided to compare prediction accuracies as a function of the number of predictions relative to the total number of contacts in the protein. In particular, we compare predictions for different proteins at the same *sensitivity*, i.e. the fraction of all true contacts that are predicted.

**Figure 7:**
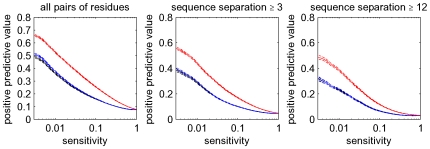
Accuracy of contact predictions for all 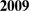 alignments. Shown are the performances of mutual information (black), 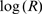 (blue), and the posterior probabilities (red). The vertical axis shows mean positive predictive value (PPV, solid line) plus and minus one standard error (dashed lines) as a function of sensitivity (horizontal axis, shown on a logarithmic scale). The left panel shows predictions for all residue pairs, the middle using only predictions for residues separated by at least 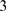 positions in the primary sequence, and the right panel for pairs separated by at least 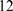 positions.

As mentioned previously, 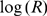 values typically increase with the number of sequences in the alignment and also depend on the phylogenetic distances of the sequences present in the alignment, such that 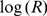 values cannot be directly compared across different domains. Therefore, for each domain we produced three lists of predicted edges, one sorted by mutual information, one by 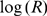, and one by posterior probability 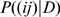. For different fractions 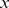, we selected the top edges from each list such that the fraction of all true edges among the predictions (sensitivity) equals 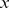, separately for each domain. For each value of 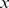 and all three measures, we then calculated the average positive predictive value, i.e. the fraction of all predicted edges that are truly in contact in the three-dimensional structure of the domain, by averaging over all domains. These results are shown in the left panel of Figure 7.

Not surprisingly, residues that are close in the primary sequence are much more likely to contact each other in the structure than distant pairs, see [20] and figure 11 below. In particular, residues that are neighbors in the primary sequence are (by the definition used) *always* contacts and residues at distance 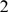 are contacting almost 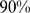 of the time, whereas contacts between residues more distal in the primary sequence are relatively rare. Therefore, if one considers all contacts, the accuracy of the predictions is dominated by the large number of contacts between residues at primary sequence distances 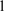 and 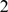, which almost always exist, and are therefore not informative regarding protein structure. Therefore, the middle panel of Figure 7 shows the results when considering only pairs that are at least 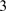 residues apart in primary sequence. In addition, following the practice established in the contact prediction literature, we also show results when considering only pairs at least 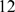 residues apart in primary sequence (Figure 7, right panel) and at least 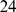 residues apart (Figure S2).

As expected, the accuracy of predictions for mutual information and 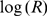 are very similar and demonstrate that these two measures can be considered equivalent in this context (we will only refer to 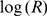 from hereon). Most importantly, Figure 7 shows that the predictions based on posterior probabilities (red curves) outperform the other methods by a large margin, i.e. with an almost 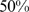 larger PPV at some sensitivities. This confirms that rigorous treatment of indirect dependencies strongly improves contact predictions. It should be noted, however, that at cut-offs where the positive predictive value is reasonably high, sensitivities are only on the order of one percent. It is thus clear that at high PPV, our method in its current form can only predict a minor fraction of all true interacting pairs, which is in accordance with results from previous studies [10],[14].

For completeness, we also considered the accuracy of prediction that would be obtained if, instead of summing over all possible spanning trees, we determine the maximum-likelihood tree and use only the links in this tree in our predictions, i.e. as done in [15]. As shown in Figure S3, although this leads to an improvement over using 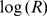, the accuracy of the posterior probability measure by far outperforms the predictions based on the maximum-likelihood tree. This nicely demonstrates the value of summing over all possible spanning trees which is employed in the calculation of the posterior for a given edge.

### The posterior removes indirect dependencies and predicts contacts with weaker statistical dependency

**Figure 8:**
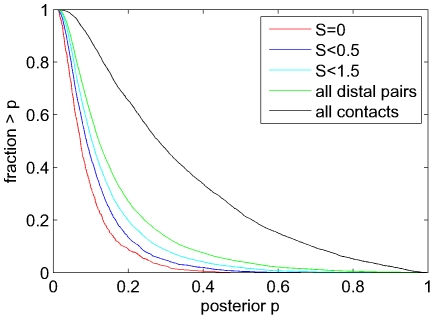
Posteriors reflect the extent to which co-evolving pairs can be explained by contact chains. Shown are the reverse cumulative distributions of the posteriors of distal co-evolving pairs (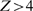) that can be explained by contact chains of scores 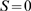 (red), 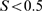 (dark blue), 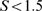 (light blue), and for all distal co-evolving pairs (green). For comparison the reverse cumulative distribution of posteriors for co-evolving contacts (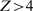) is also shown (black).

To demonstrate that our model successfully prevents the prediction of interactions between pairs with indirect dependency, we collected all distal pairs that showed significant statistical dependence (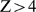) and ordered them by the score of the best co-evolving contact chain that can explain their statistical dependency, i.e. as shown in Figure 4. Figure 8 shows the reverse-cumulative distributions of the posteriors that these distal pairs obtain in our model for different cut-offs on the best path score 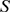, as well as the distribution of posteriors of all contacting pairs with 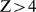.

First of all, we see that co-evolving contacts have dramatically higher posteriors than distal pairs in general, which confirms the improved accuracy of contact predictions that our method accomplishes. Moreover, we see that distal pairs that can be explained with the most strongly co-evolving contact chains, i.e. with the lowest scores 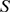, obtain the lowest posterior probabilities. For example, less than 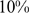 of the distal pairs with a chain at score 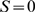 have a posterior larger than 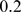 and virtually no pair has a posterior as large as 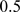. As the score 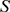 of the best chains increases, so generally do the posteriors. This confirms that the posterior as calculated by our model correctly captures the extent to which a statistical dependency is direct.

Instead of selecting all distal co-evolving pairs with contact chains below some score 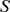, we also selected all co-evolving pairs with 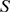 scores larger than various cut-offs and determined the distributions of their posteriors. These distributions are shown in Figure S4 and illustrate that distal co-evolving pairs with sufficiently large score 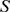 obtain posteriors comparable with those of co-evolving contacts. This suggests that the particular subset of distal co-evolving pairs that cannot be explained by any chain of contacts are likely true interacting residues, which may for example form contacts in the interaction surface of oligomers of the domain.

**Figure 9:**
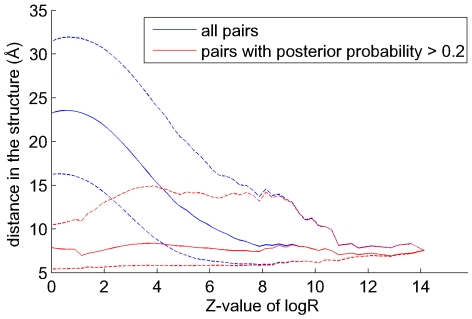
The posterior predicts structurally close pairs independent of their direct statistical dependence. The structural distance distribution (vertical axis) is shown for all pairs (blue) and for pairs with posterior probability larger than 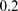 (red) as a function of the 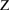-value of the 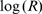 statistic (horizontal axis). The solid lines show the medians of the distributions and the dashed lines the 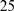
th and 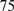
th percentiles.

To further demonstrate that our Bayesian network model correctly distinguishes direct from indirect interactions, we also investigated the extent to which the posterior identifies structurally close pairs independent of the direct statistical dependency of the pair. We divided all pairs into bins according to their 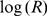 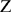-value and calculated, for each bin, the distribution of structural distances of all pairs, and for the subset of pairs that have posterior probability larger than 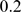. Figure 9 shows, as a function of the 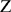-value of the pairs, the median, 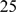
th, and 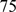
th percentiles of the structural distance distributions of all pairs (blue) and those with posterior larger than 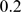 (red).

At large 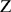-values the red and blue curves are essentially identical. In this regime, we are only looking at the most strongly dependent residues in each alignment and any spanning tree of high likelihood must contain edges between these pairs of residues, i.e. almost all of these edges have high posterior probabilities. However, already at 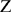-values as high as 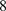, the median distance of all pairs starts to increase rapidly, from roughly 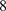
Å to more than 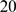
Å at 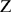-value 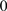. This illustrates again that even at very high values of 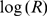 a substantial fraction of pairs are distal in the structure. In contrast, the subset of residues with high posterior probability remains close over the whole range of 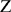-values, down to 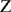-values of almost 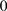. In fact, strikingly, there is very little change in the distribution of structural distances for 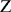-values from 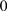 to 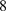. This is very significant because it demonstrates that, independent of the amount of direct statistical dependency between a pair of positions, a high posterior is indicative of close structural distance. Moreover, it demonstrates that our Bayesian network model can detect truly interacting pairs of residues even if they show only a small amount of statistical dependency.

### The Bayesian network model with phylogenetic correction significantly outperforms existing methods

One of the key problems in contact prediction is the large number of distal pairs with high statistical dependency. In the foregoing sections we have shown that many of these distal co-evolving pairs are indirect, induced by chains of dependencies between contacting residues, and we have shown that our Bayesian network model can rigorously disentangle direct from indirect dependencies, thereby greatly improving contact predictions. In the remaining sections we develop a number of extensions of our basic method to further improve the predictions.

As mentioned in the introduction, the phylogenetic relationships of the underlying sequences is a major confounding factor when determining the statistical dependency between several residues (nicely explained in eg [9],[13]) and it is a difficult task to ‘subtract’ from the apparent statistical dependency between two residues the part that is purely due to phylogeny. The best way to address this difficulty would of course be to construct a phylogenetic tree of all sequences in the multiple alignment and to explicitly model the evolution of the sequences along the tree, using an evolutionary model that takes dependencies between positions into account. Unfortunately, it appears that such a rigorous approach is computationally intractable for several reasons. First, one would either have to accurately reconstruct the phylogenetic tree, which is very challenging for large sets of sequences, or sum over all possible trees, which is computationally infeasible. The second issue is the evolutionary model. In our Bayesian network model, the conditional probabilities 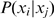 are different at every pair 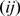, introducing 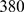 parameters per pair, which are integrated over. However, for the evolutionary case analytic integration is no longer possible, which makes such models intractable. Indeed, models that treat dependencies between residues in an explicit phylogenetic setting [12],[15] consider much simpler evolutionary models in which only correlations in the overall *rates* of mutations at different positions are considered and not the specific identities of the mutations.

As an alternative to explicit phylogenetic methods, recently a number of simple *ad hoc* phylogenetic corrections have been proposed, which do not involve a reconstruction of the phylogenetic tree, which can be efficiently calculated, and which clearly improve contact predictions [13],[14]. One of these corrections, the so-called *average-product correction* APC has been shown to provide the most accurate contact predictions [14]. It is based on the idea that the statistical dependency between every pair of columns is the sum of a true statistical dependency and a background dependency due to the phylogenetic relationships. In the APC it is assumed that the background dependency is a product of independent factors associated with the two positions. Since a given position will interact with only a small fraction of other positions, the background dependencies can be estimated by calculating, for each column, its average statistical dependence with all other columns. The background dependence for each pair is then subtracted to obtain a corrected statistical dependency. As described in *Materials and Methods*, we adapted the APC to our Bayesian model, essentially replacing 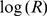 with a corrected version 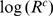 that subtracts out the background dependency. These 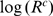 values can then be used, analogously to 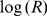 values, to determine corrected posterior probabilities (see *Materials and Methods*).

**Figure 10:**
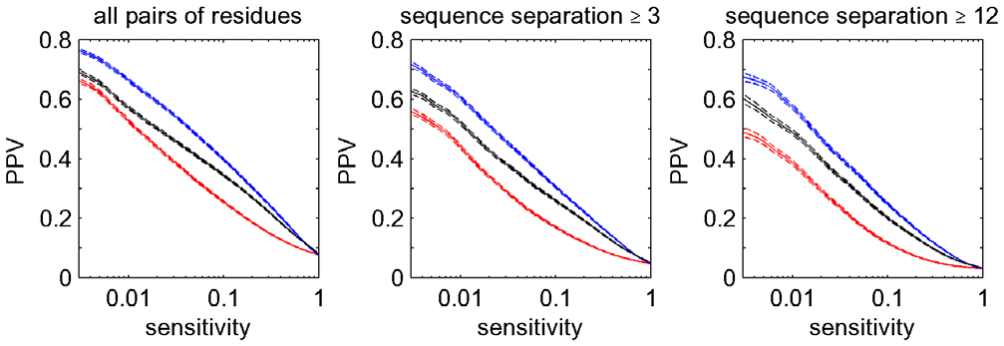
Improved accuracy of contact predictions when a phylogenetic correction is included. In blue, we show the performance of the phylogenetically-corrected posterior probabilities, in black the performance of the predictions based on the average-product corrected (APC) mutual information [14], and in red the performance of the posterior probability without phylogenetic correction. Curves were calculated as in figure 7.

In figure 10, we show the accuracy of our predictions using the corrected posterior probabilities (in blue) and compare it with predictions based on mutual information using the average-product correction APC (in black). The latter has been recently shown to outperform other existing methods [14]. The red curves show the performance of the method without the phylogenetic correction, i.e. as was shown in Figure 7. It is clear that the predictions based on posterior probability combined with the phylogenetic correction significantly outperform the current best methods. For example, considering pairs at primary sequence separation at least 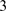, the sensitivities at PPV of 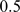 are 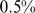 for the uncorrected posterior, about 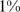 for the APC, and about 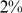 for the corrected posterior. The clear improvement in prediction accuracy is also evident for pairs with primary sequence separation of at least 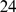 amino acids (Figure S5).

Although Figure 10 combines results of the predictions on protein domains of differing sizes, the fact that the true interactions are a much smaller fraction of all possible interactions for long sequences makes the prediction task significantly harder for long sequences, see e.g. [29]. In Figure S6, S7, S8, and S9, we show the performance of the various methods separately for short, medium length, and long sequences. We find that, independent of the length of the sequences, our method clearly outperforms current methods.

### Co-evolution of residue pairs is independent of primary sequence separation

**Figure 11:**
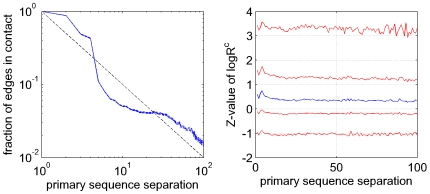
Occurrence of contacts and co-evolution as a function of primary sequence separation. Left panel: The fraction of residue pairs that are in contact in the structure as a function of primary sequence separation 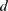. The solid blue line shows the mean, the dashed blue lines the mean 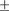 one standard error. The dashed black line shows the function 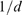. Right panel: The 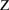-value distribution of the 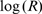 statistics for all contacting pairs at different primary sequence separations. The blue line represents the median and the red lines represent the 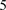
th, 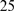
th, 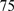
th and 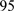
th percentiles, respectively. The 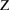-value was calculated with respect to the mean and standard deviation of the 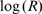 distribution of all pairs (including distal ones). In both panels only sequence separations up to 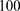 residues are shown as the curves become very noisy for larger sequence separations.

In protein structure prediction, where prediction of contacts at large sequence separations is particularly important [21], it is well-known that contact prediction accuracy generally decreases with increasing sequence separation ([20],[21], also seen in figure 10). This is a direct consequence of the fact that the fraction of contacts decreases rapidly as a function of sequence separation (roughly as 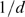, where 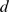 is the primary sequence separation, see the left panel in figure 11), which makes the prediction problem much more difficult for contacts at large primary sequence separations. Vice versa, because contacts at large primary distances are rare, they are most informative for protein structure prediction [21].

The left panel of Figure 11 shows that there are several regimes in the distribution of contact-density at different primary sequence distances. First, residues at distance 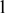 and 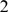 are almost always contacts and thus contain very little information about protein structure. In contrast, at distances 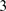 and 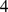 the fraction of contacts has already dropped to roughly 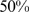, i.e. about 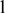 bit of information per contact, and the fraction then drops quickly, reaching about 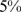 at primary sequence separation 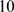. For distances between 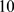 and 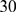 the fraction stays roughly constant at 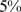 and for even larger distances it drops approximately as 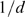.

Clearly, the information contained in Figure 11 regarding protein structures can be used to improve contact prediction, i.e. by assigning prior probabilities to different contacts based on their distance in primary sequence. However, before pursuing this we ask to what extent contacts at different primary sequence distances show statistical evidence of co-evolution. The almost ubiquitous contacts at primary sequence distances 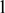 and 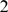 are probably mainly the result of geometrical constraints, the contacts at intermediate distances are likely often part of the same secondary structure, and the very distal contacts might correspond to contacts between different secondary structure elements. Given the different nature of these contacts at different primary sequence separations, one might expect very different distributions of statistical dependencies, and this would clearly affect contact prediction.

To investigate this, we determined the distribution of the 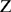-values of corrected 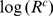 for all *contacts* at each primary sequence separation 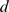 (Figure 11, right panel). Interestingly, the distribution of statistical dependencies is almost *constant* across the entire range of primary sequence distances. The only significant deviation is a slight peak at sequence separation 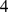, corresponding to residues on the same side of alpha helices ([30] and data not shown), which apparently have slightly increased statistical dependency compared to other contacts. However, far more important for the purpose of predicting protein structure is that, with regard to the statistical dependency between alignment columns, all contacts appear to be essentially equal, so that the evidence of statistical dependency between residues can be treated completely independently of the prior information regarding which contacts are more or less likely to exist based on general structural considerations. From a biological and evolutionary perspective this result shows that, interestingly, different ‘types’ of contacts apparently lead to similar evolutionary constraints.

### Influence of entropy on contact prediction

An important, but poorly understood issue in covariation-based contact prediction is the influence of conservation on prediction accuracy. The ‘conservation’ shown by a position in a multiple alignment can be most generally quantified by the entropy of the amino acid distribution in the column. It is well known that this column entropy can vary immensely along protein sequences, most probably due to functional and structural constraints. One would intuitively expect that a position that is contacting many other residues would generally have to satisfy more constraints and would thus be expected to show relatively low entropy.

**Figure 12:**
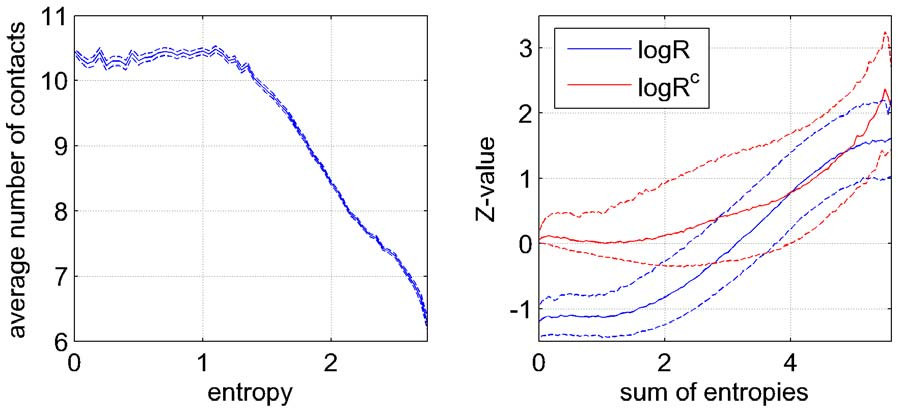
Contact-degree and co-evolution as a function of positional entropy. Left panel: Average number of contacts of a residue (solid line) as a function of the entropy of its alignment column. The dashed lines denote mean 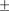 one standard error. The right panel shows the Z-value distribution of both 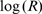 (blue) and 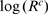 (red) for all contacting pairs versus the sum of entropies of the corresponding columns. The solid lines denote the medians and the dashed lines the 25th and 75th percentiles.

To investigate this, we calculated, for each position in each domain, the column entropy and the number of contacts of the corresponding residue. As shown in the left panel of Figure 12 there is indeed a clear negative correlation between the column entropy and the number of contacts. For very low entropies, i.e. less than 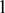, the average number of contacts is constant and approximately 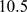. As the entropy increases from 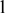 to about 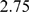 (which is close to the entropy of a uniform distribution of amino acids) the average number of contacts drops to almost 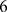. That is, very low entropy columns have on average almost twice as many contacts as high entropy columns. Since the number of residues in a sphere of 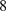
Å around the 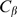 atom of an amino acid (which is exactly our definition of a contact) is commonly used as a measure for how strongly a residue is buried in the core of the protein (e.g. [31]), the left panel of Figure 12 reiterates the well-known dependence between surface accessibility and conservation [32].

It is well appreciated in the literature that the variation of entropy across positions has important effects on predictions based on statistical dependencies. For example, a comparative study of different prediction methods has shown that commonly used co-variation measures differ in their sensitivity to per-site variability and generally, each method has highest accuracy within its specific preferred range of variability [10]. In analogy to our analysis of statistical dependency as a function of distance in primary sequence (Figure 11, right panel), we investigated how the statistical dependency that different contacts exhibit depends on the column entropies of the residues. As before, we transformed the 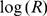 values to 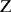-values and determined the 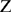-value distribution of all contacts as a function of the sum of the entropies of the corresponding columns (Figure 12, blue lines). We see that contacts indeed show a strong correlation between the sum of column entropies and statistical dependency. For low entropy columns the 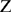-values are mostly negative, and they become only positive at an entropy sum of about 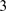. It is thus clear that contact predictions that use mutual information (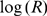) will preferentially predict contacts between residues of high entropy columns.

That mutual information and 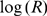 is low for contacts with low entropy columns is to a certain extent unavoidable. It is a basic result of information theory [17] that the mutual information between two variables cannot be larger than the minimum of the marginal entropies of the two variables. Intuitively, one could imagine a position that is so constrained by its function and its many contacts that only a single amino acid is viable at the position. Obviously, since this position shows no variation whatsoever it cannot display any signs of statistical dependency with any other column, even though it may contact many other residues. This is a basic limitation of using statistical dependency for contact prediction that cannot be avoided. However, it has been argued that modified versions of mutual information, such as the product or sum correction [14], besides correcting for the phylogenetic background signal, are also able to better identify co-evolution between less variable residues. The red lines in the right panel of Figure 12 show the mean and standard deviation of the 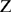-values of product-corrected statistical dependency 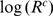. We see that indeed, the correlation between the 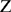-values and the sum of column-entropies is significantly reduced when using 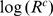, and low entropy contacts no longer show negative 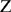-values on average.

Still, a clear correlation between the column-entropy sum and the statistical dependency remains even for 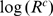. On the one hand this may be the result of the inherent inability to ‘detect’ statistical dependency when columns are very conserved. On the other hand, it is also conceivable that those positions that have low entropy, and that form many contacts, may generally show weaker statistical dependency *per contact*. For example, it could be argued that hydrophobic residues that lie in the core of the protein and thus contact many other residues are less variable because they need to remain on the interior and therefore do not allow for changes towards non-hydrophobic residues. Such residues may not be constrained so much by their contacting residues, but rather by the necessity to stay away from the solvent-exposed protein surface, leading to relatively weak statistical dependencies with the contacting residues.

### Incorporation of prior information improves prediction accuracy

So far our Bayesian method assumes that a contact between any pair of positions is a priori equally likely. However, as seen in the previous sections, the probability for a contact to occur depends strongly on the primary sequence distance between the residues and the column-entropies of the residues. We therefore developed an ‘informative prior’ which makes the prior probability for a contact to occur depend on both of these variables. For a given pair of positions, let 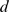 be the distance in the primary-sequence of the two positions, and let 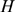 denote the sum of the column-entropies of these positions. As described in *Materials and Methods*, we estimated the fractions 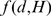 of pairs at sequence distance 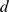 and entropy-sum 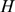 that are contacts and using these fractions constructed prior probability distributions that can be easily incorporated into our method.

**Figure 13:**
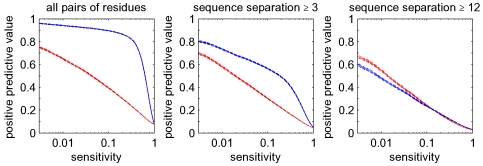
Improved accuracy of contact prediction when an informative prior is included. In blue, we show the performance of the posterior probabilities that take primary-sequence separation and column entropy into account. For comparison we show in red the performance of the posteriors with phylogenetic correction but uniform prior, which are the same as the blue lines in Figure 10.

Figure 13 shows the results of the contact predictions performed with our Bayesian network model incorporating the informative prior and using posterior probabilities (blue lines). For comparison the results using posteriors based on 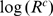 (the blue lines in Figure 10) are shown as well (red lines). We see that, for the set of all pairs, and all pairs that are at least 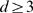 apart in primary sequence, the incorporation of the prior probability dramatically improves the predictions. For example, looking at all pairs, our method can predict roughly 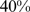 of all existing contacts at a positive predictive value of 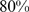. If we restrict ourselves to non-trivial contacts, i.e. those with primary-sequence distance 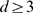, we find that at a positive predictive value of 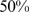 our method reaches a sensitivity of roughly 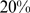. For comparison, without the prior an approximately 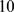 times lower sensitivity is reached at the same positive predictive value.

Somewhat surprisingly, we find that the quality of the predictions for distal pairs 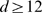 is slightly reduced by the incorporation of the prior, especially at low sensitivities. We speculate that this is a result of the fact that we constructed the prior distribution assuming that 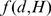 is independent of the length of the domain itself. This approximation breaks down most significantly when focusing on distal pairs because, whereas contacts at short primary distances occur in all domains, contacts at long primary distances are more common in long domains. However, it should be noted that, given that contacts at this primary-sequence distance are rare, one would most likely need to perform predictions at reasonably high sensitivity, i.e. 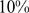 or more. In this regime, the performance with prior is comparable to or even a tiny bit better than without prior.

## Discussion

One of the key problems in using co-evolution analysis to predict residue contacts is that so many structurally distal pairs show strong statistical dependencies [14]–[16]. A number of reasons have been proposed to explain this fact. One explanation is that sequences in multiple alignments are generally phylogenetically related and these phylogenetic relationships can induce strong apparent statistical dependencies between many pairs of columns. Although there is of yet no computationally tractable way for treating the phylogenetic dependencies in a rigorous manner, i.e. by explicitly modeling the evolution of the sequences including arbitrary dependencies, several procedures have been proposed that can correct at least for the main phylogenetic signal [8],[9],[14],[15]. Indeed the application of such methods has been shown to very significantly improve contact predictions [9],[14],[15].

Still, even with the current best phylogenetic corrections, strong statistical dependencies remain evident between many structurally distal pairs. One proposed explanation that has received little attention in the contact prediction literature is that statistical dependencies between distal pairs can be induced by the percolation of statistical dependencies along chains of co-evolving contacts [23],[24]. Here we have shown that such chains of co-evolving contacts are indeed pervasive across all protein domains and that they explain many if not most of the distal co-evolving pairs. Statistical analysis shows that these chains travel on average 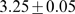
Å per contact, and that the total distance covered by these chains is exponentially distributed with an average of 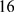
Å, corresponding to a chain that consists of 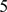 contacts. Note that, whereas residues up to 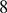
Å apart are generally considered contacts, our results strongly suggest that the typical distance between co-evolving contacts is only 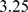
Å. Another interesting observation is that, although it is likely that contacts between residues at different distances in primary sequences are different in nature, our analysis shows that the statistical dependency shown by contacts is completely independent of their primary-sequence separation. This is an important insight because it demonstrates that co-evolutionary analysis is equally informative about close and distal contacts.

We have adapted our recently evolved Bayesian network model [27] in order to assign, to any pair of positions, a posterior probability that they interact directly. This posterior probability rigorously takes into account all possible ways in which the statistical dependence between the pair can be explained in terms of chains of other co-evolving pairs. Analysis of the predictions of this model shows that it correctly detects distal pairs that can be explained by co-evolving contact chains, and that it also allows one to detect true interacting pairs that have only weak direct statistical dependency.

Recently Halabi et al [33] have shown that, by a spectral analysis of the matrix of statistical dependencies between positions, one can identify so called ‘protein sectors’: sets of positions that co-evolve significantly with each other, but that are relatively independent of the positions in other sectors. Since in [33] a rather simple measure of direct statistical dependency is used, we speculate that a much more accurate identification of protein sectors could be obtained by using statistical dependencies as assessed by our posterior probabilities.

While finishing the work in this study, a paper appeared that also aims to disentangle direct from indirect interactions [22]. Like our approach, [22] models the joint probability of sequences in the multiple alignment in terms of a set of pairwise interactions. What is appealing about the approach of [22] is that it is based on the more ‘physical’ assumption that an interaction energy is associated with each pairwise interaction such that a total interaction energy can be calculated for each sequence, and that the probability to observe a particular sequence is given simply by the Boltzmann distribution in terms of this total energy. However, the great disadvantage of this model is that its solution requires a heuristic approximation and is computationally very expensive to calculate. For example, in [22] the authors were forced to restrict themselves to only 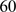 positions in the alignment, and even then the calculations for a single alignment took several days. Therefore, an application of the approach of [22] on as large a scale as in this work, with thousands of multiple alignments of up to several hundred positions, is not feasible. In addition, it is not clear how the approach of [22] could accommodate a phylogenetic correction, which would be necessary to obtain a competitive performance with this method.

Although the disentangling of direct and indirect statistical dependencies strongly improves contact predictions, and incorporating a phylogenetic correction further improves the performance, the predictions are still far from perfect. In particular, at reasonably high positive predictive value the sensitivity amounts to less than 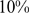 of all true contacts. Although it is clear that contact predictions based only on statistical dependencies could be further improved, for example by a more rigorous treatment of the phylogenetic dependencies, we believe that it is unlikely that such improvements would dramatically enhance the performance. First of all, simple inspection of the data shows that a large number of the pairs that are contacts in the sense that they are less than 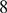
Å apart, really show no sign of co-evolution at all. That is, a large fraction of ‘contacts’ may simply not interact directly, and these obviously can never be detected using statistical dependence measurements. On the other end of the scale are residues that contact so many others that they are very strongly constrained, and show almost no variability in evolution. For such highly conserved residues it is also inherently impossible to identify their interaction partners using co-evolutionary analysis.

We thus believe that the largest further improvements to contact prediction are to be expected from incorporating information other than statistical dependency. To illustrate that additional information can be easily incorporated into our model, we developed an informative prior that takes into account that the likelihood of a contact to exist depends on the primary-sequence distance of the residues, and that highly conserved residues tend to have a higher number of contacts. The incorporation of even this simple additional information already leads to dramatic improvements in contact prediction. Clearly more powerful priors could be developed that take into account more sophisticated structural knowledge. In addition, in our current method we integrate over all possible joint probabilities for pairs of interacting residues, effectively assuming that all possible joint probability distributions are equally likely. Here too improvements could likely be made by taking into account prior knowledge on which joint probability distributions are more or less likely for interacting pairs of amino acids. Ultimately the most satisfying approach would be to combine our approach with direct structural modeling, i.e. somewhat along the lines of the approach taken in [34].

Following the plausible intuition that, the more different kinds of information are taken into account, the greater the prediction accuracy that can be obtained, several machine learning and statistical methods have been proposed that incorporate a much larger number of different features (see [20],[34],[35] and references therein). Besides primary sequence separation and conservation, these methods include features such as domain length, relative solvent accessibility, predicted secondary structure, the amino acid composition in short windows around the positions of interest, chemical properties of the amino acids, and contact potentials. Due to varying training and test sets and varying standards of evaluation, it is very difficult to compare the performance of our method with these approaches. However, some principal differences between these methods and ours should be noted. First, all these methods rely on training sets to fit parameters, so that additional methods are required to avoid over-fitting, whereas our method is essentially without any tunable parameters and does not require any training sets. Second, some of these methods are rather *ad hoc* ‘black box’ methods, e.g. neutral networks [20] or support vector machines [35], that use partially redundant sets of features, from which it is typically hard to derive mechanistic insights. In contrast, our method is derived directly from first principles. In any case, the results that we have presented show that it is crucial to take indirect dependencies into account when incorporating co-evolution information. We have provided a rigorous method for doing so and it is clear that any contact prediction method that incorporates co-evolution information would strongly benefit from using our method for disentangling direct and indirect dependencies.

Whereas we have here applied our method to predict contacting residues in a single protein, it is straight forward to use the same method for predicting contacting residues between pairs of proteins that are known to interact. That is, given two set of orthologs proteins 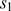 and 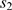, for which it is known that each member of set 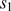 interacts with the corresponding member of set 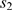, we can simply concatenate the multiple alignments of 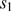 and 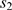 into one longer multiple alignment, and apply our method to this longer alignment.

More generally, our method provides a computationally tractable extension of weight matrix models to take into account arbitrary pairwise dependencies, and there are a number of more general applications that we envisage pursuing in the future. First, our method can be generally used to ‘score’ multiple alignments in a way that includes pairwise dependencies. This could be used to discover subfamilies within large multiple alignments or to generally refine multiple alignments. Since the performance of alignment-based contact prediction methods is expected to depend strongly on the quality of the alignments, such a refinement may further improve contact prediction. Finally, another attractive application is to develop a regulatory-motif finding algorithm that takes into account arbitrary pairwise dependencies between positions.

## Materials and Methods

### Domain sequences and structures

Domain alignments and the mappings from domains to available structures in the PDB database were downloaded from the Pfam database [19],[36]. We only used Pfam A, which is the high-quality and manually curated part of Pfam [19]. For each Pfam domain with at least one known structure, we reduced the alignment to positions corresponding to match states of the corresponding Pfam hidden Markov model with no more than 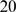 percent gaps. The removal of columns with many gaps is necessary as gaps can cause spurious correlations (see below) and make it difficult to compare the phylogenetic background signal between different columns. We removed from each alignment all multiple copies of identical sequences as well as sequences that had more than 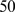 percent gaps with respect to the match states. Additionally, alignments containing less than 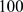 sequences or less than 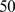 columns were discarded. To keep computational times limited we also removed alignments with more than 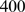 columns. For each Pfam alignment, all corresponding PDB files were collected according to the iPfam annotation [36] and distances between pairs of residues were determined as the distance between the 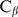 atoms (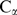 for glycines). In the case of NMR models, the minimal distances of all models contained in the PDB entry were chosen. If a Pfam domain was present in multiple protein structures or in several chains of one protein structure, we chose the median distance over all chains and structures. For some alignments the corresponding structure did not cover all columns in the alignment and we discarded the small number of examples where the coverage was less than 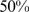. This resulted in 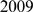 domains with structurally-defined distances between residues. Finally, distance in primary sequence was defined as the distance between the match states of the alignment.

### Probabilistic model

Our Bayesian network model was described in detail in [27]. Briefly, given a single column 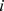 of the alignment with observed amino acid counts 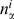, the probability 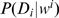 of the column is given in terms of the (unknown) probability distribution 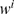, with 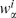 the probability that letter 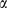 occurs at position 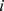, i.e. 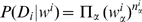. Using a Dirichlet prior for 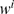 with parameter 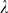, we obtain the marginal probability of the column 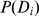 by integrating over all possible distributions 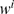. This integral can be performed analytically and the result can be expressed in terms of gamma functions:
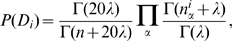
(2)where 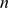 is the number of sequences in the alignment. Similarly, the *joint* probability of the data 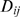 in a pair of columns 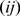 is given in terms of the number of times 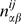 that the combination of letters 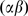 occurs at positions 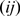, i.e.
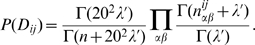
(3)Here, we set the parameter 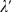 of the Dirichlet prior for the joint probability distribution to 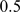. As shown in [28], in the context of a dependence tree model, consistency requires that 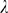 equals 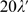.

The statistical dependence between columns 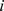 and 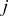 is quantified by the ratio
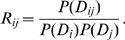
(4)The connection of 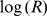 to mutual information is easily established by substituting equations (2) and (3) into the logarithm of 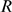 as given by (4) and using Stirling's approximation to the logarithm of the gamma function. We then find that approximately
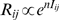
(5)for large 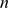, with 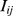 the mutual information between columns 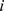 and 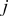. Importantly, when determining the counts 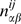 and 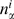 in order to determine 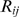, we discard all pairs of residues within a given sequence where either 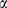 or 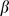 is a gap. Treating gaps as a 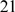 amino acid causes strong spurious correlations between residues that are close in primary sequence since gaps usually come in blocks (data not shown).

A *dependence tree* 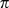 specifies for each position 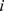 (except for the root of the tree) a parent position 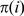 which is the residue that 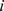 depends on. To keep the notation simple, we here use the symbol 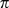 to both denote the mapping from a node to its parent node and the dependence tree itself. It can be shown [27] that, given a dependence tree, the joint probability 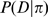 of the entire alignment can be written as
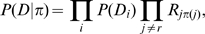
(6)where the first product goes over all positions and the second over all positions except for the root 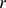.

Finally, the probability 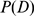 of the whole alignment is given by summing over all possible dependence trees 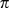


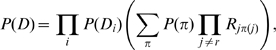
(7)where 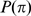 is the prior probability of a particular spanning tree 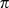. The last product is in fact the product of the 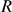-values over all edges of the tree given by 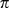 and is independent of the choice of the root. If the prior probability of a spanning tree can be written as a product of probabilities 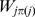 along each edge 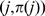 of the tree
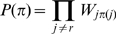
(8)then equation (7) can be rewritten as
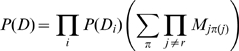
(9)with 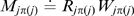. Thus, the weight of each edge is simply multiplied by its prior probability. The largest term in the sum of equation (9) is the *maximum spanning tree* when a weight 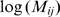 is assigned to each edge 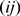 and this maximum spanning tree can be easily determined [37].

The sum over spanning trees in (9) can be calculated using a generalization of Kirchhoff's matrix-tree theorem [28]. For this we need to calculate the Laplacian of the matrix 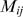, which is defined as
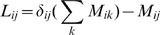
(10)where the sum goes over all columns (or rows) of the 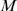-matrix and 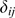 is the Kronecker delta function, which is one if 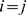 and zero otherwise. We can then write the sum over all spanning trees as
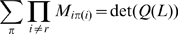
(11)where 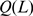 is the matrix 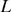 with one line and column removed (the determinant is independent of which line and column are removed). The summation over all spanning trees (there are 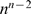 spanning trees for a full graph with 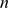 nodes) thus reduces to the calculation of a determinant, which can be done in a time proportional to 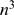.

As discussed previously [27], the calculation of the determinant of the matrix 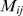 is numerically very challenging since the entries 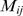 vary over many orders of magnitude. In order to circumvent this problem, we rescale the entries of the matrix as suggested in [38]:
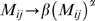
(12)with 
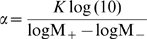
 and 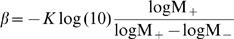 where 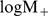 (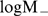) is the logarithm of the maximal (minimal) entry of the matrix 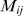. This function maps all 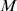 values into the interval 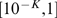, preserves the relative ordering of entries and does not exaggerate relative differences in belief [38]. The lower bound 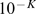 ensures that the rescaled 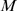-matrix remains numerically non-singular. 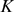 can be set according to the numerical precision of the machine and we set 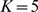. We then use these rescaled 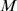-values to calculate the posterior probabilities.

### Calculating posteriors

Using expression (7), the posterior probability of a particular edge 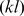 is given by
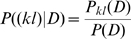
(13)where
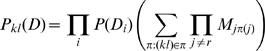
(14)which is the sum of the probabilities 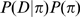 for all spanning trees 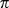 that contain the edge 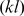. This expression can be calculated by replacing the set of 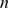 nodes with a set of 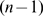 nodes, in which nodes 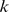 and 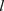 are contracted to one node, say 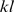, and the edge weights of this new node 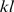 are given by 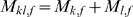 for all nodes 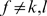 [39]. Using this construction we can write the sum over all spanning trees containing edge 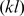 as
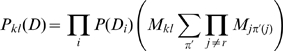
(15)where the sum now goes over all spanning trees 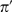 of the 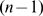 nodes. This sum over spanning trees can of course also be calculated as a determinant as described above. Roughly speaking, an edge 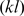 will have high posterior if it occurs in the large majority of all spanning trees 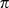 that have high probability 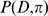.

### Phylogenetic correction

Due to the phylogenetic relatedness of the sequences in the alignment, there typically will be a statistical dependence between residues even in the absence of a functional linkage of these positions. Previous work [14] showed that this dependence can be corrected for (to some extent) by assuming that, due to phylogenetic relationships, each position has a certain amount of ‘background’ statistical dependence with other columns. Since each position interacts only with a small fraction of all other positions this background dependence can be estimated by calculating the average mutual information of that position with all the remaining positions. In [14], two types of corrections were proposed, a multiplicative one, named APC, and a additive one, named ASC. We here briefly review the derivation of these corrections.

The idea of the ASC is that the mutual information 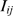 between positions 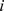 and 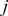 is the sum of the true mutual information 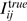 and background mutual informations 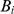 and 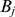, associated with positions 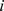 and 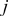, i.e.
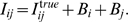
(16)We define average mutual informations as
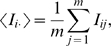
(17)with 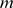 the number of columns of the alignment. Other averages like 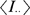, 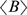, and so on, are defined analogously. Note that, for notational simplicity, in these averages we have adopted the convention that 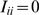. We can then derive the equalities
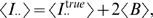
(18)and
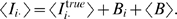
(19)If one assumes that, since true interactions are relatively rare, the averages 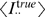 and 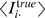 are much smaller than 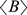, we can set 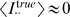 and 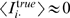 and have
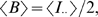
(20)and
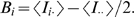
(21)Finally, under these assumptions the true mutual information 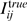 is then given by
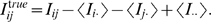
(22)Motivated by this derivation, the ASC is defined as
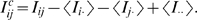
(23)


In the product correction APC we assume that the background mutual information between 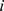 and 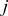 can be written as a *product* of contributions of the two columns, i.e.
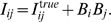
(24)Assuming again that the true average mutual informations are small we find
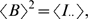
(25)and
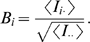
(26)Using this the APC version of the mutual information is given by
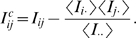
(27)


Since the APC performs better than the ASC we focused on adapting the APC for our Bayesian model. As mentioned above, the logarithms of the 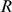 values are the equivalent of mutual information in our model. Therefore, naively we would simply replace 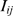 with 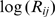 in equation (27) above. However, whereas the mutual information naturally has a lower bound of zero, which is reached only for independent positions, 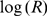 is off-set with respect to mutual information and becomes *negative* for independent positions. Note also that all posterior probabilities are invariant under a global shift of all the 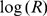 values by a constant. Therefore, we substitute into equation (27) a shifted version of 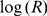 which is guaranteed to be non-negative. For each domain we determine the minimal value 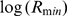 and define a shifted version of 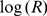 as
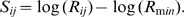
(28)Using these shifted 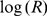
s we then define the corrected 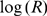 as
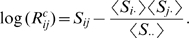
(29)In our model with phylogenetic correction we simply replace each factor 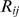 with 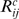.

### Prior probability of spanning trees

**Figure 14:**
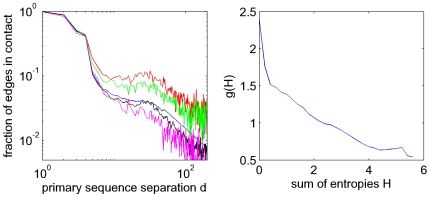
Estimation of prior probabilities. The left panel shows the dependence between the fraction of pairs that are in contact and primary sequence separation for all pairs (in blue) as well as for pairs whose sum of entropies lies in a given entropy bin (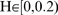 in red, 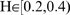 in green, 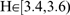 in black and 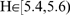 in magenta). For the sake of clarity, only a few selected entropy bins across the entire range are shown. The right panel shows the estimated function 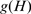, which describes how the probability of an edge to be a contact depends on the sum of entropies of the corresponding columns of the alignment (see text).

Our Bayesian model easily allows for the incorporation of prior probabilities on each spanning tree via the edge probabilities 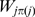 in equation (9). Here, we use these edge probabilities to include the dependence on both the primary sequence separation of the positions in the pair (Figure 11), as well as the sum of the entropies of the corresponding columns (Figure 12). To estimate the fraction 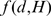 of all pairs with sequence-separation 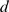 and entropy-sum 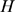 that are contacts, we separated all pairs of columns into entropy bins of width 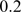, spanning the whole range of entropies 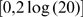 and compared the dependence on primary sequence separation within the different bins (Figure 14, left panel).

We see that, irrespective of the column entropy sum 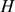, the fraction 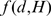 has approximately the same shape as a function of 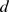 as the overall fraction of contacts 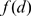 which we showed in Figure 11. We find that for distances 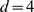 or less the fraction is virtually independent of entropy, i.e. 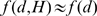, while for larger distances the fractions 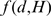 are roughly proportional to 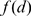, with a proportionality constant that decreases with entropy 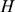. That is, we assume the following general form for 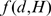:
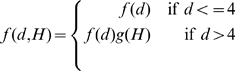
(30)


We first estimated 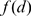 directly from the observed fractions as shown in Figure 11 for all sequence separations up to 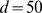. As 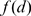 is proportional to 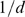 for sequence separations 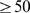 and becomes very noisy for large sequence separations (data not shown), we approximate the curve as 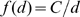 for sequence separations 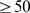 (blue line in Figure 14). The constant 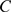 is chosen so that the curve is continuous at 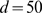. We then determined the function 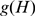 by numerically maximizing, for each fixed entropy bin 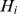, the likelihood of the data, which is given by
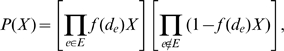
(31)where the first product runs over all edges 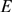 with 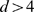 and 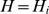 that are contacts, the second product over all edges with 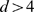 and 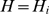 that are not contacts, and 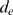 stands for the primary sequence separation of edge 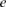. The value 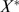 that maximizes the likelihood of the data determines the value of 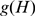 for the bin 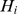, i.e. 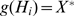. The resulting function 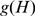 is shown in the right panel of figure 14. Clearly the probability of an edge decreases with the entropy-sum 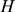, i.e. it drops by almost a factor of 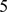 from the lowest to the highest entropy edges.

Finally, in order to assign prior probabilities to different possible spanning trees, we assume a random graph model where each edge 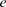 occurs with a probability 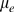 that is proportional to 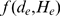, with 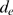 the primary sequence separation, and 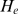 the entropy sum of edge 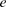. Note that each spanning tree only contains 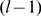 edges for a domain of length 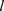, and we thus have to ensure that our random graph model produces on average 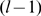 edges. As the expected number of edges in a random graph is equal to the sum over all 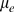, we set 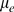 to
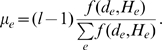
(32)


Let 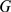 be the full graph including all 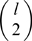 edges of a particular domain and let 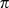 be one particular spanning tree 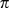. We can now write the prior probability of the tree as
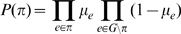
(33)Here, the first product runs over all edges 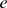 in the tree 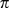 and the second one over all edges in 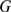 that are not in the tree 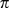. Since the posteriors are independent of a global rescaling of all prior probabilities 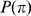, we divide 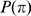 by the probability of the graph that contains no edges, to obtain
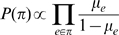
(34)which is independent of the edges that are not contained in the tree. We can thus set the edge weights 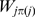 in equation 9 to
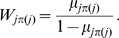
(35)Unfortunately, we cannot directly used 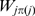 to calculate the matrix entries 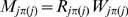 in equation 9. As discussed above, the 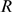-values relate to mutual information 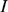 through 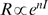, where 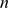 is the total number of sequences in the alignment. However, even when the phylogenetic correction is employed, because the 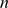 sequences contain many phylogenetically closely-related sequences, the number of *statistically independent* sequences is generally much smaller than 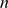. Because of this, even the corrected 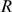-values still significantly overestimate statistical dependence. To take this into account we define the matrix entries 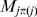 as
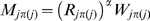
(36)where 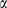 is a free parameter, which must lie between 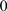 (only prior information) and 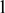 (original 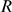-values). Note that, through this transformation, we are assuming that instead of 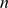 independent sequences, there are only 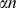 effectively independent sequences. The PPV-sensitivity curves for varying values of 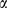 are shown in Figures S10, S11, and S12. For the curve in the main text, we chose 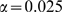, so as to maximize the accuracy for pairs with 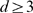 without a significant decrease in accuracy for pairs with 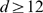.

## Supporting Information

Figure S1 Number of contacts *n* versus the number of residues *l* per protein domain for varying separations in primary sequence. The red lines are the regression lines (in log-space), corresponding to the power-laws *n = 2.43l^1.12^*, *n = 0.16l^1.43^* and *n = 0.05l^1.62^*. The dashed black line corresponds to *n = l*.
(0.33 MB TIF)

Figure S2 Accuracy of contact predictions for all 2009 alignments based on mutual information (black), log*(R)* (blue), and posterior probabilities (red). For different values of sensitivity, the corresponding number of predictions for each domain and each method were selected and their positive predicted value (PPV), i.e. the fraction of correct predictions, was calculated (vertical axis). Dashed lines indicate mean PPV plus/minus one standard error. The top left panel shows predictions for all residue pairs, the top right one using only predictions for residues separated by at least 3 positions in the primary sequence, the bottom left one for pairs separated by at least 12 positions, and the bottom right panel for pairs separated by at least 24 positions.
(0.32 MB TIF)

Figure S3 Comparison of prediction accuracy for log*(R)* (blue), for the log*(R)* values contained in the maximum-likelihood tree (green) and for the posterior probability (red). As the maximum-likelihood tree only predicts *l-1* edges, where *l* is the number of columns of the alignment, the different measures cannot be directly compared in terms of sensitivity (there would be finite-length effects as predictions by the maximum-likelihood tree measure cannot reach a sensitivity of *1*). Instead, we sort the predictions per domain and, for each fixed cut-off on the rank *r*, we show the average positive predictive value (solid lines) for all predictions with rank *r* or higher. The dashed lines indicate plus/minus one standard error. As the shortest domains in our dataset have length 50, all domains are included in the calculation of the green curve for ranks 1 to 49. The blue and green curves are identical for high ranks as all the highest-scoring edges are included in the maximum spanning tree. However, for decreasing ranks, the maximum-spanning tree discards edges that can be explained indirectly, which leads to an improvement in performance. Importantly, the posterior probability significantly outperforms the maximum-spanning tree predictions both for low *and* high ranks.
(0.36 MB TIF)

Figure S4 Posteriors reflect the extent to which co-evolving pairs can be explained by contact chains. Shown are the reverse cumulative distributions of distal co-evolving pairs (Z*>4*) that cannot be easily explained by contact chains, i.e. where the best scoring chain has a score of *S>2* (red), *S>3* (dark blue), or *S>4* (light blue). For comparison the reverse cumulative distributions of posteriors for all co-evolving distal pairs (green) and all co-evolving contacts (black) are also shown.
(0.13 MB TIF)

Figure S5 Accuracy of contact predictions for all alignments. In blue, we show the performance of the phylogenetically-corrected posterior probabilities, in black the performance of the predictions based on the average-product corrected (APC) mutual information, and in red the performance of the posterior probabilities without phylogenetic correction. Curves were calculated as described in the main text.
(0.33 MB TIF)

Figure S6 Accuracy of contact predictions for alignments of length 50 to 100. In blue, we show the performance of the phylogenetically-corrected posterior probabilities, in black the performance of the predictions based on the average-product corrected (APC) mutual information, and in red the performance of the posterior probabilities without phylogenetic correction. Curves were calculated as described in the main text.
(0.33 MB TIF)

Figure S7 Accuracy of contact predictions for alignments of length 101 to 200. In blue, we show the performance of the phylogenetically-corrected posterior probabilities, in black the performance of the predictions based on the average-product corrected (APC) mutual information, and in red the performance of the posterior probabilities without phylogenetic correction. Curves were calculated as described in the main text.
(0.33 MB TIF)

Figure S8 Accuracy of contact predictions for alignments of length 201 to 300. In blue, we show the performance of the phylogenetically-corrected posterior probabilities, in black the performance of the predictions based on the average-product corrected (APC) mutual information, and in red the performance of the posterior probabilities without phylogenetic correction. Curves were calculated as described in the main text.
(0.33 MB TIF)

Figure S9 Accuracy of contact predictions for alignments of length 301 to 400. In blue, we show the performance of the phylogenetically-corrected posterior probabilities, in black the performance of the predictions based on the average-product corrected (APC) mutual information, and in red the performance of the posterior probabilities without phylogenetic correction. Curves were calculated as described in the main text.
(0.33 MB TIF)

Figure S10 Accuracy of contact predictions including the informative prior for different values of the weighting parameter *α*, including the limit of using only the informative prior (*α* = *0*). The positive predictive value (vertical axis) is shown as a function of sensitivity (horizontal axis). Different colors correspond to different values of *α* (see legend) and dashed lines show mean plus and minus one standard error. For comparison, we also show the performance of the posterior when using no prior information (black). Note that the horizontal axis is shown on a logarithmic scale.
(0.37 MB TIF)

Figure S11 Accuracy of contact predictions including the informative prior for different values of the weighting parameter *α*, including the limit of using only the informative prior (*α* = *0*), when considering only pairs that are at least *d = 3* apart in primary sequence. The positive predictive value (vertical axis) is shown as a function of sensitivity (horizontal axis). Different colors correspond to different values of *α* (see legend) and dashed lines show mean plus and minus one standard error. For comparison, we also show the performance of the posterior when using no prior information (black). Note that the horizontal axis is shown on a logarithmic scale.
(0.36 MB TIF)

Figure S12 Accuracy of contact predictions including the informative prior for different values of the weighting parameter *α*, including the limit of using only the informative prior (*α* = *0*), when considering only pairs that are at least *d = 12* apart in primary sequence. The positive predictive value (vertical axis) is shown as a function of sensitivity (horizontal axis). Different colors correspond to different values of *α* (see legend) and dashed lines show mean plus and minus one standard error. For comparison, we also show the performance of the posterior when using no prior information (black). Note that the horizontal axis is shown on a logarithmic scale.
(0.33 MB TIF)
