# Supplementary figures and images for: Disentangling Direct from Indirect Co-Evolution of Residues in Protein Alignments
Source: PLoS Comput Biol. 2010 Jan 1;6(1):e1000633. doi: 10.1371/journal.pcbi.1000633 (PMC2793430; doi:10.1371/journal.pcbi.1000633)

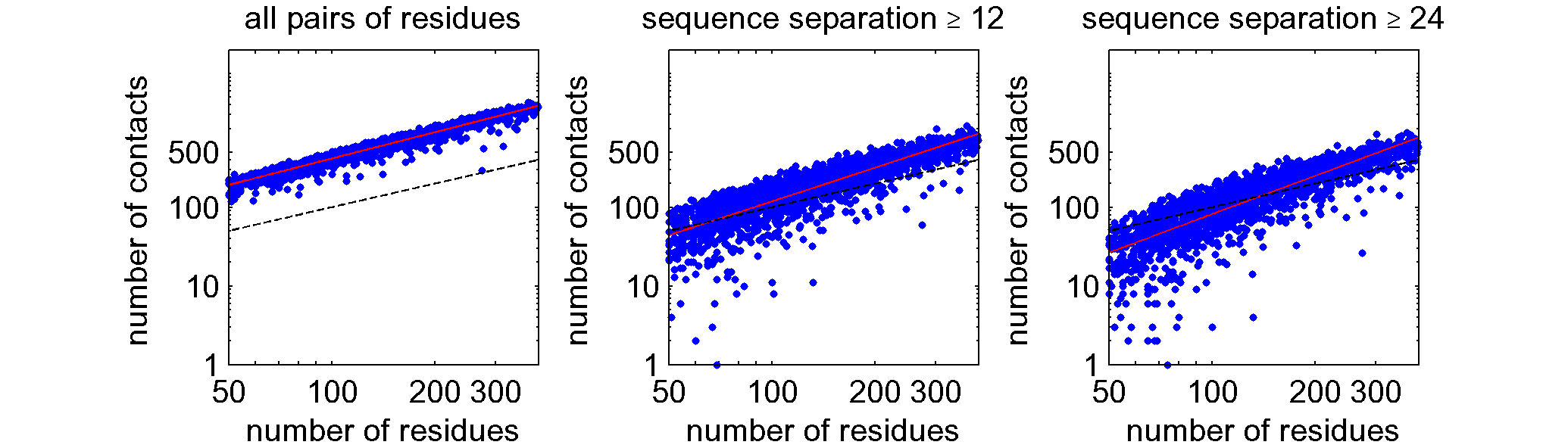

Supplement: Figure S1 — Number of contacts n versus the number of residues l per protein domain for varying separations in primary sequence. The red lines are the regression lines (in log-space), corresponding to the power-laws n = 2.43l1.12, n = 0.16l1.43 and n = 0.05l1.62. The dashed black line corresponds to n = l. (0.33 MB TIF) [file pcbi.1000633.s001.tif]

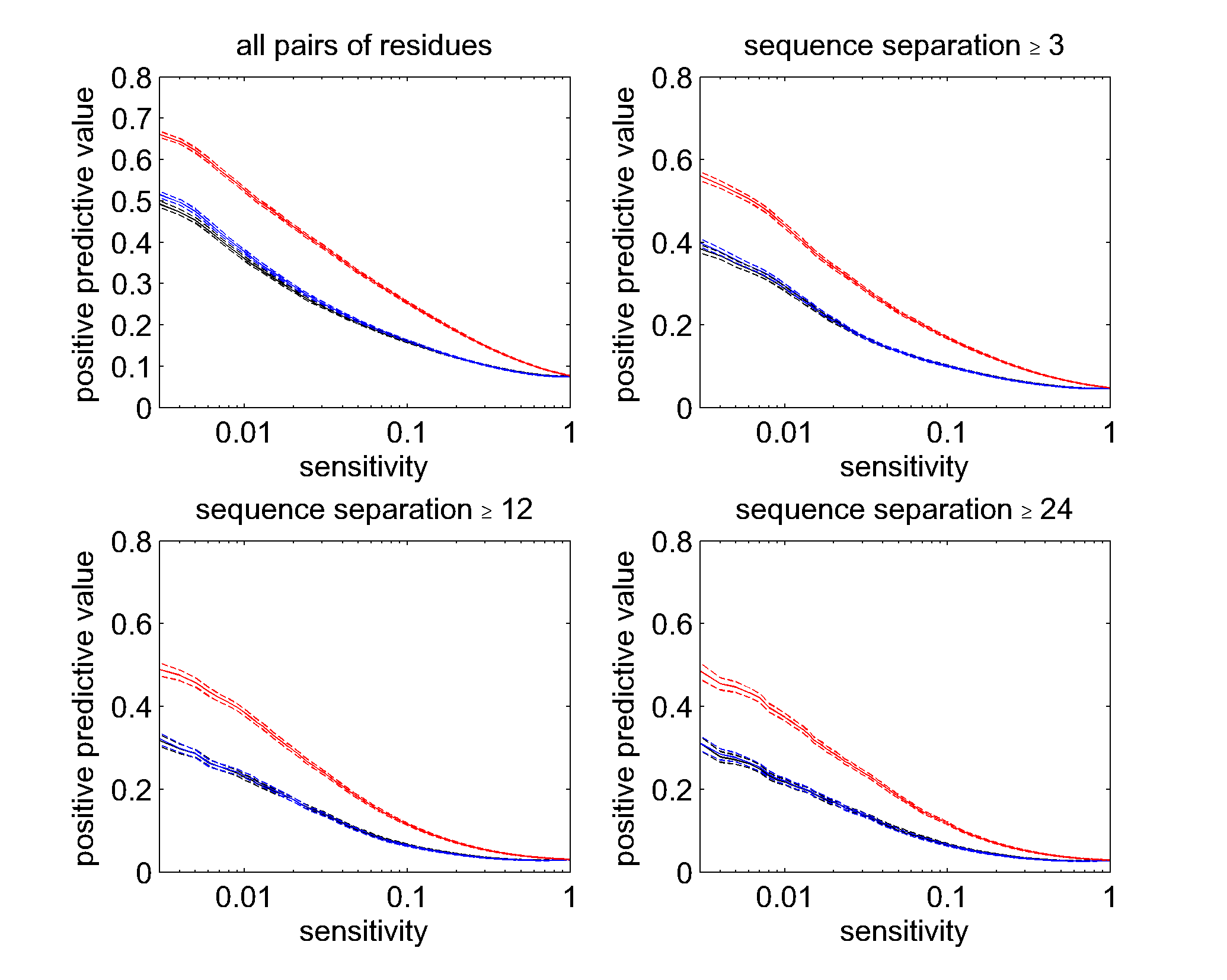

Supplement: Figure S2 — Accuracy of contact predictions for all 2009 alignments based on mutual information (black), log(R) (blue), and posterior probabilities (red). For different values of sensitivity, the corresponding number of predictions for each domain and each method were selected and their positive predicted value (PPV), i.e. the fraction of correct predictions, was calculated (vertical axis). Dashed lines indicate mean PPV plus/minus one standard error. The top left panel shows predictions for all residue pairs, the top right one using only predictions for residues separated by at least 3 positions in the primary sequence, the bottom left one for pairs separated by at least 12 positions, and the bottom right panel for pairs separated by at least 24 positions. (0.32 MB TIF) [file pcbi.1000633.s002.tif]

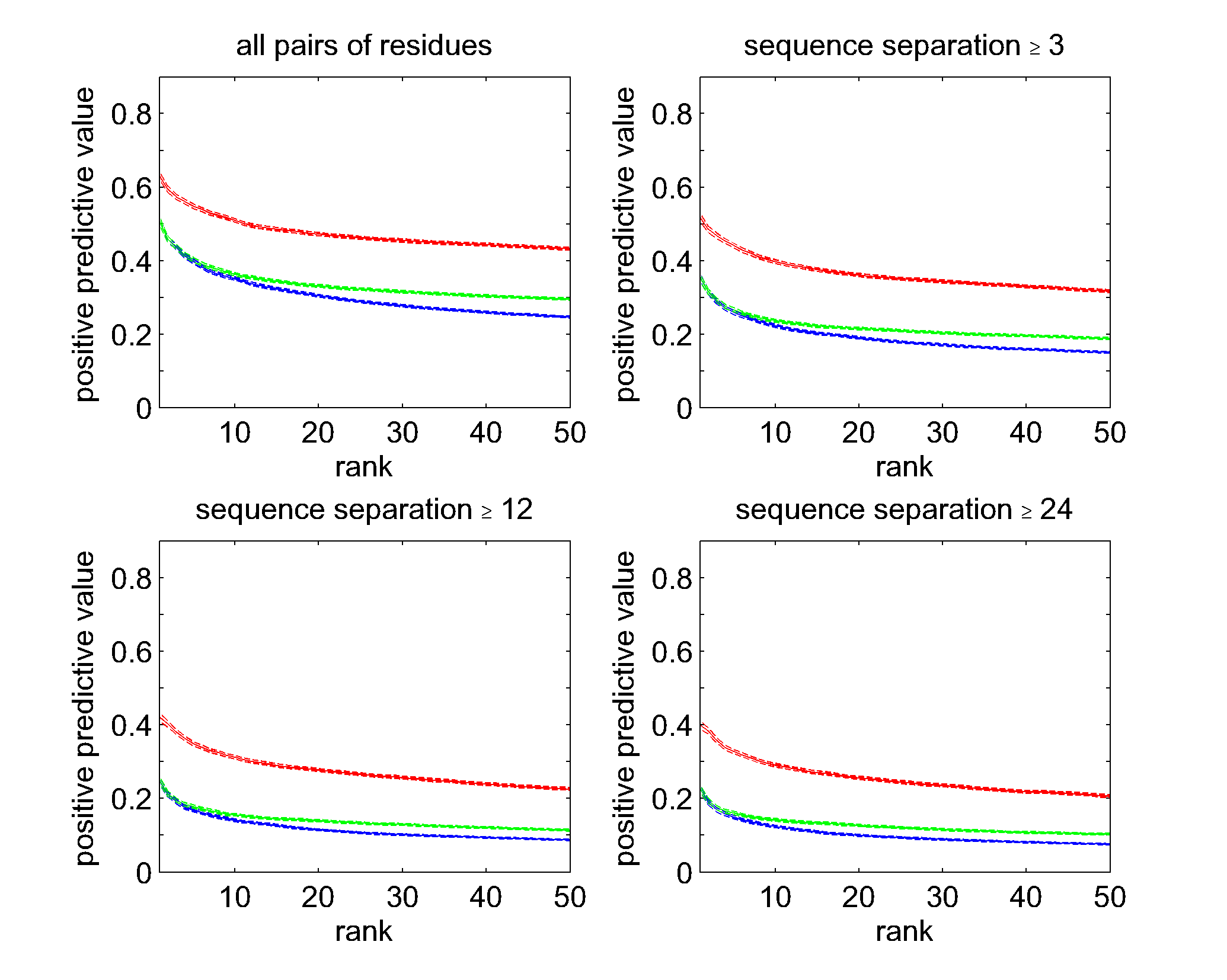

Supplement: Figure S3 — Comparison of prediction accuracy for log(R) (blue), for the log(R) values contained in the maximum-likelihood tree (green) and for the posterior probability (red). As the maximum-likelihood tree only predicts l-1 edges, where l is the number of columns of the alignment, the different measures cannot be directly compared in terms of sensitivity (there would be finite-length effects as predictions by the maximum-likelihood tree measure cannot reach a sensitivity of 1). Instead, we sort the predictions per domain and, for each fixed cut-off on the rank r, we show the average positive predictive value (solid lines) for all predictions with rank r or higher. The dashed lines indicate plus/minus one standard error. As the shortest domains in our dataset have length 50, all domains are included in the calculation of the green curve for ranks 1 to 49. The blue and green curves are identical for high ranks as all the highest-scoring edges are included in the maximum spanning tree. However, for decreasing ranks, the maximum-spanning tree discards edges that can be explained indirectly, which leads to an improvement in performance. Importantly, the posterior probability significantly outperforms the maximum-spanning tree predictions both for low and high ranks. (0.36 MB TIF) [file pcbi.1000633.s003.tif]

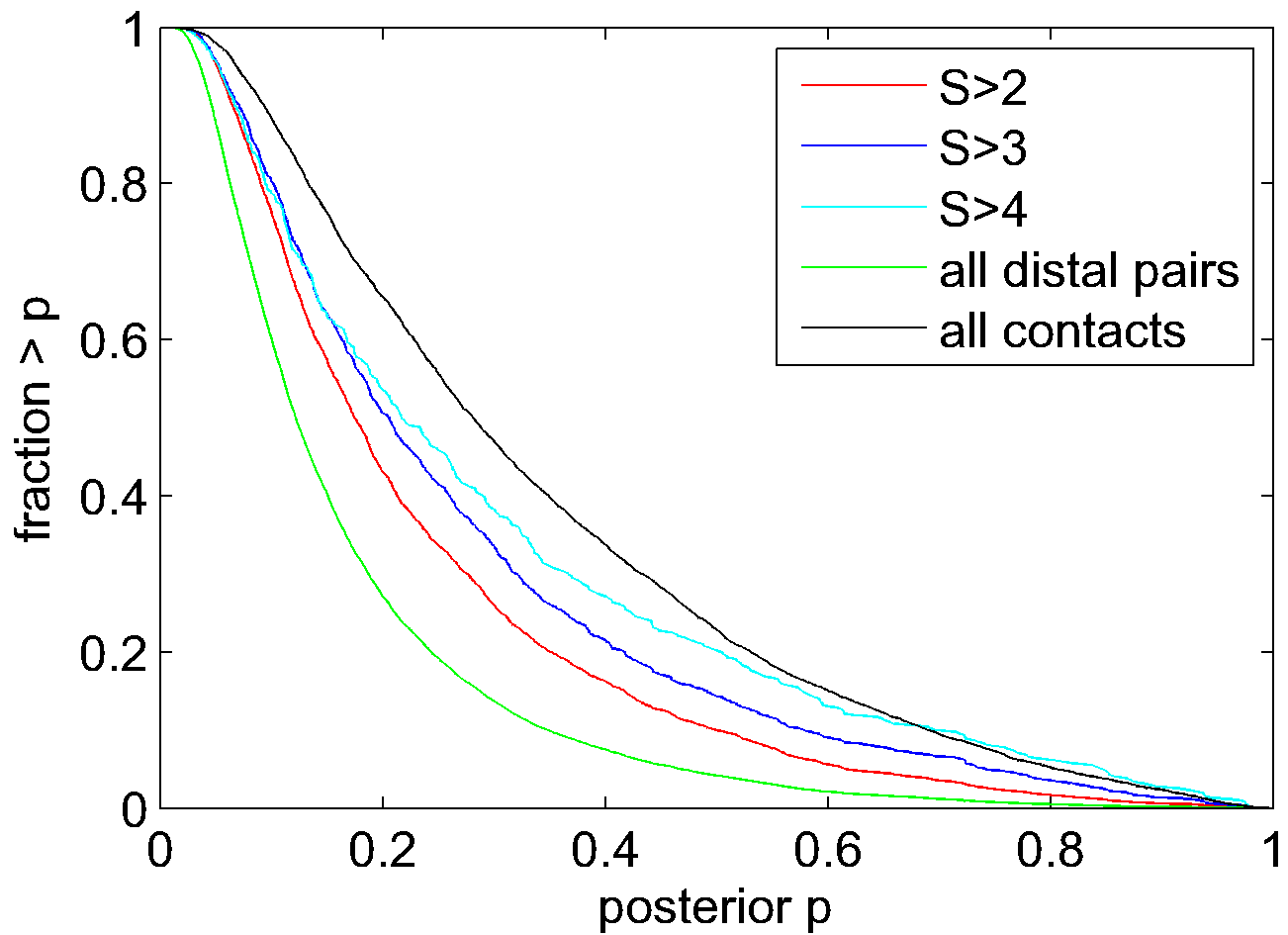

Supplement: Figure S4 — Posteriors reflect the extent to which co-evolving pairs can be explained by contact chains. Shown are the reverse cumulative distributions of distal co-evolving pairs (Z>4) that cannot be easily explained by contact chains, i.e. where the best scoring chain has a score of S>2 (red), S>3 (dark blue), or S>4 (light blue). For comparison the reverse cumulative distributions of posteriors for all co-evolving distal pairs (green) and all co-evolving contacts (black) are also shown. (0.13 MB TIF) [file pcbi.1000633.s004.tif]

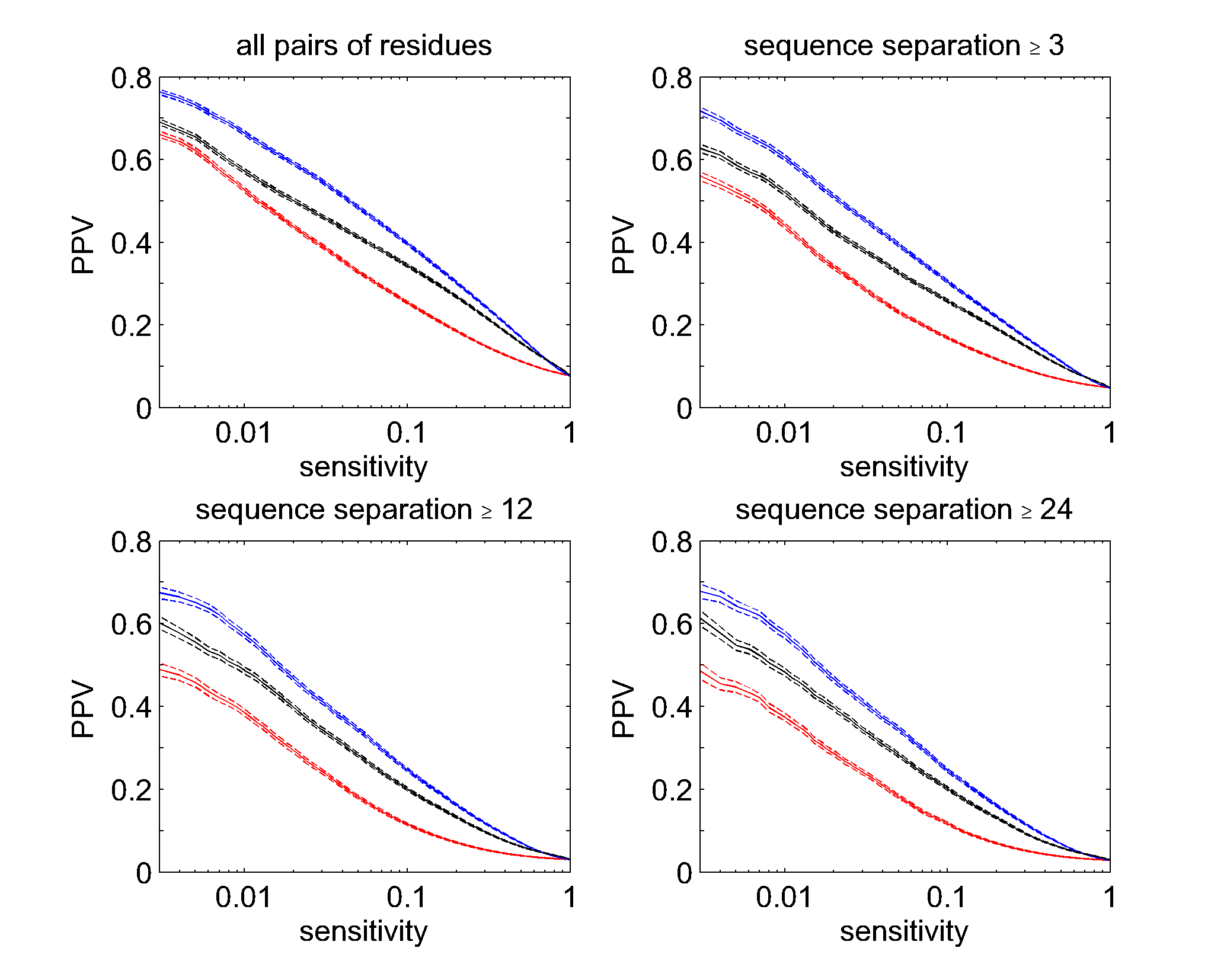

Supplement: Figure S5 — Accuracy of contact predictions for all alignments. In blue, we show the performance of the phylogenetically-corrected posterior probabilities, in black the performance of the predictions based on the average-product corrected (APC) mutual information, and in red the performance of the posterior probabilities without phylogenetic correction. Curves were calculated as described in the main text. (0.33 MB TIF) [file pcbi.1000633.s005.tif]

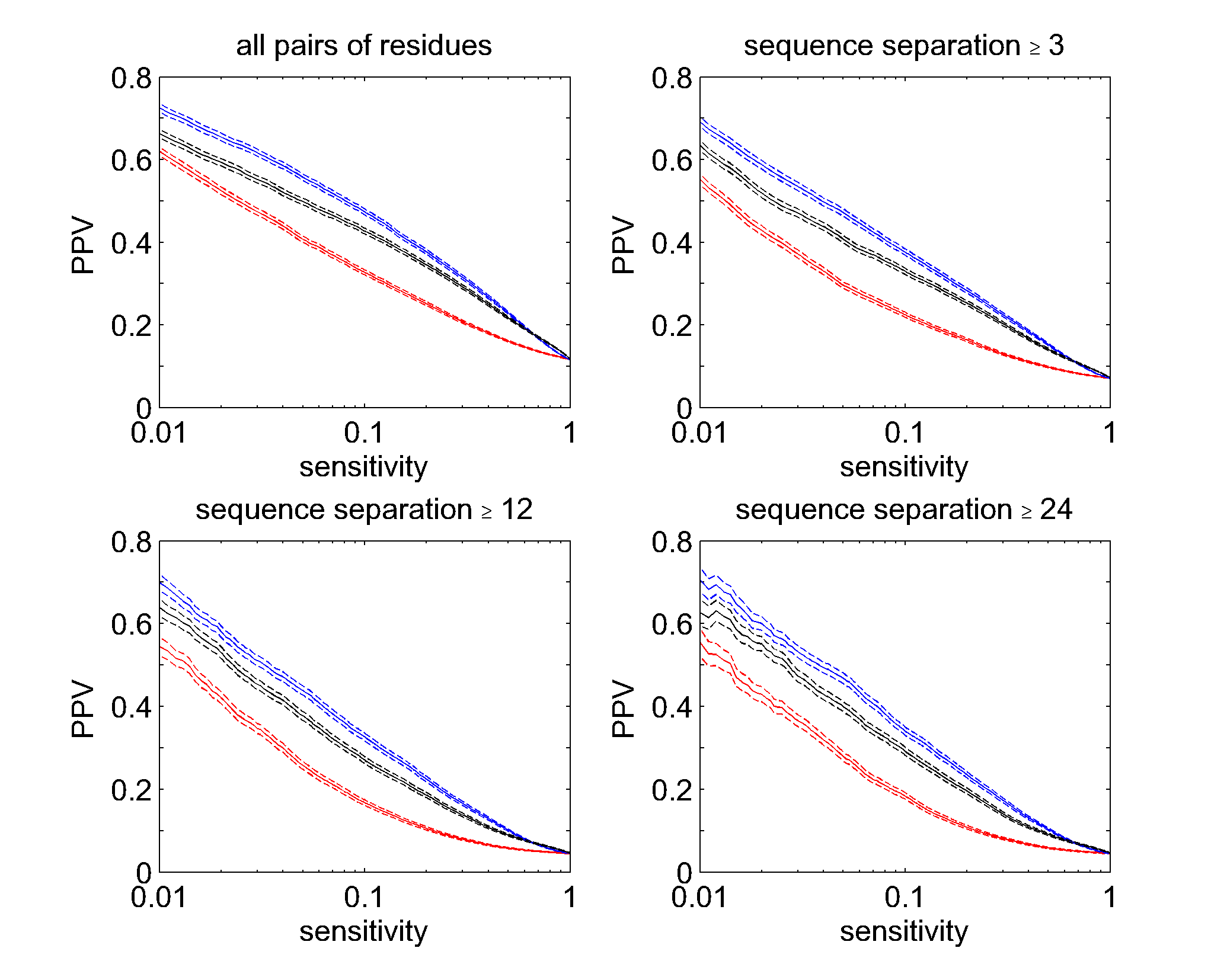

Supplement: Figure S6 — Accuracy of contact predictions for alignments of length 50 to 100. In blue, we show the performance of the phylogenetically-corrected posterior probabilities, in black the performance of the predictions based on the average-product corrected (APC) mutual information, and in red the performance of the posterior probabilities without phylogenetic correction. Curves were calculated as described in the main text. (0.33 MB TIF) [file pcbi.1000633.s006.tif]

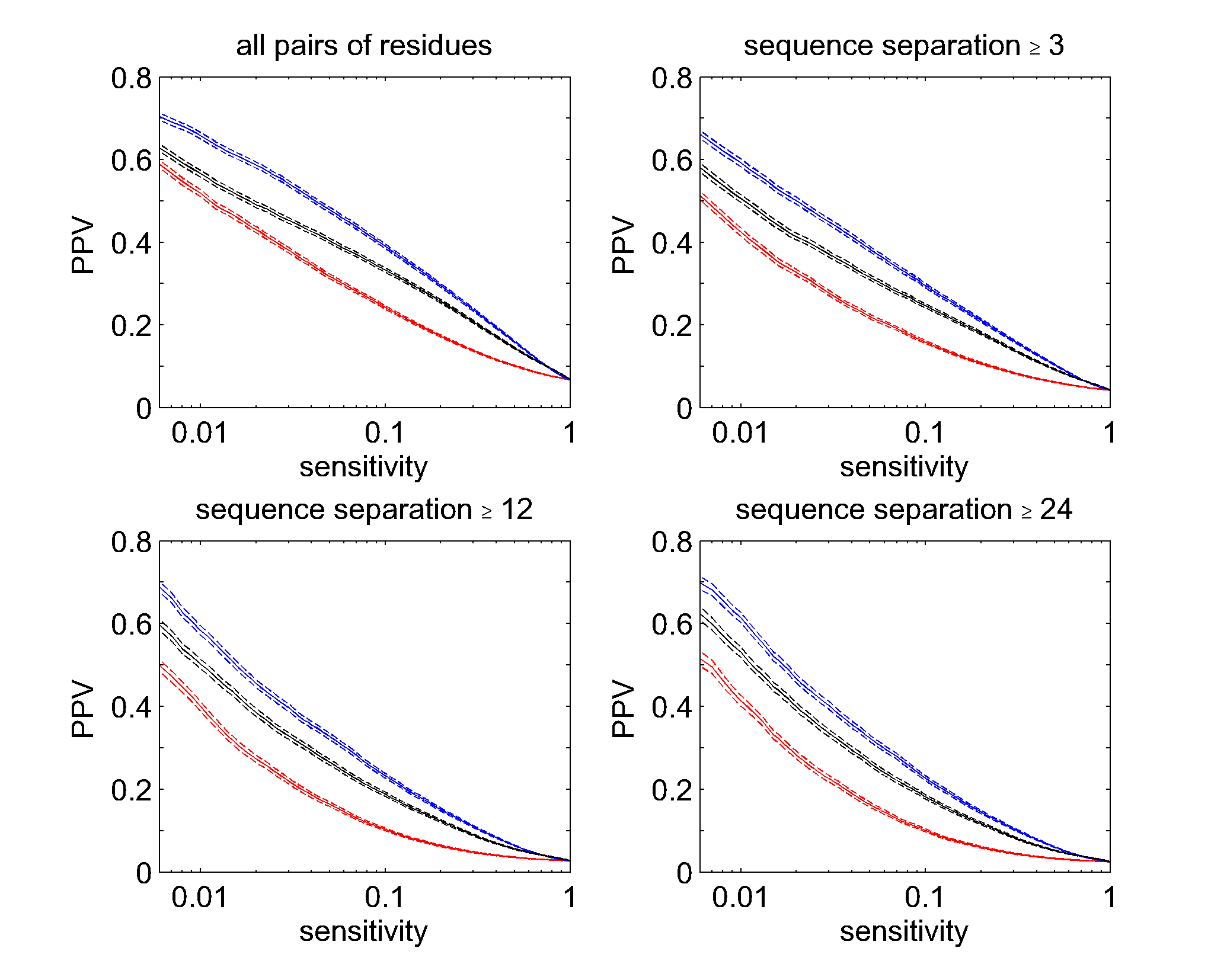

Supplement: Figure S7 — Accuracy of contact predictions for alignments of length 101 to 200. In blue, we show the performance of the phylogenetically-corrected posterior probabilities, in black the performance of the predictions based on the average-product corrected (APC) mutual information, and in red the performance of the posterior probabilities without phylogenetic correction. Curves were calculated as described in the main text. (0.33 MB TIF) [file pcbi.1000633.s007.tif]

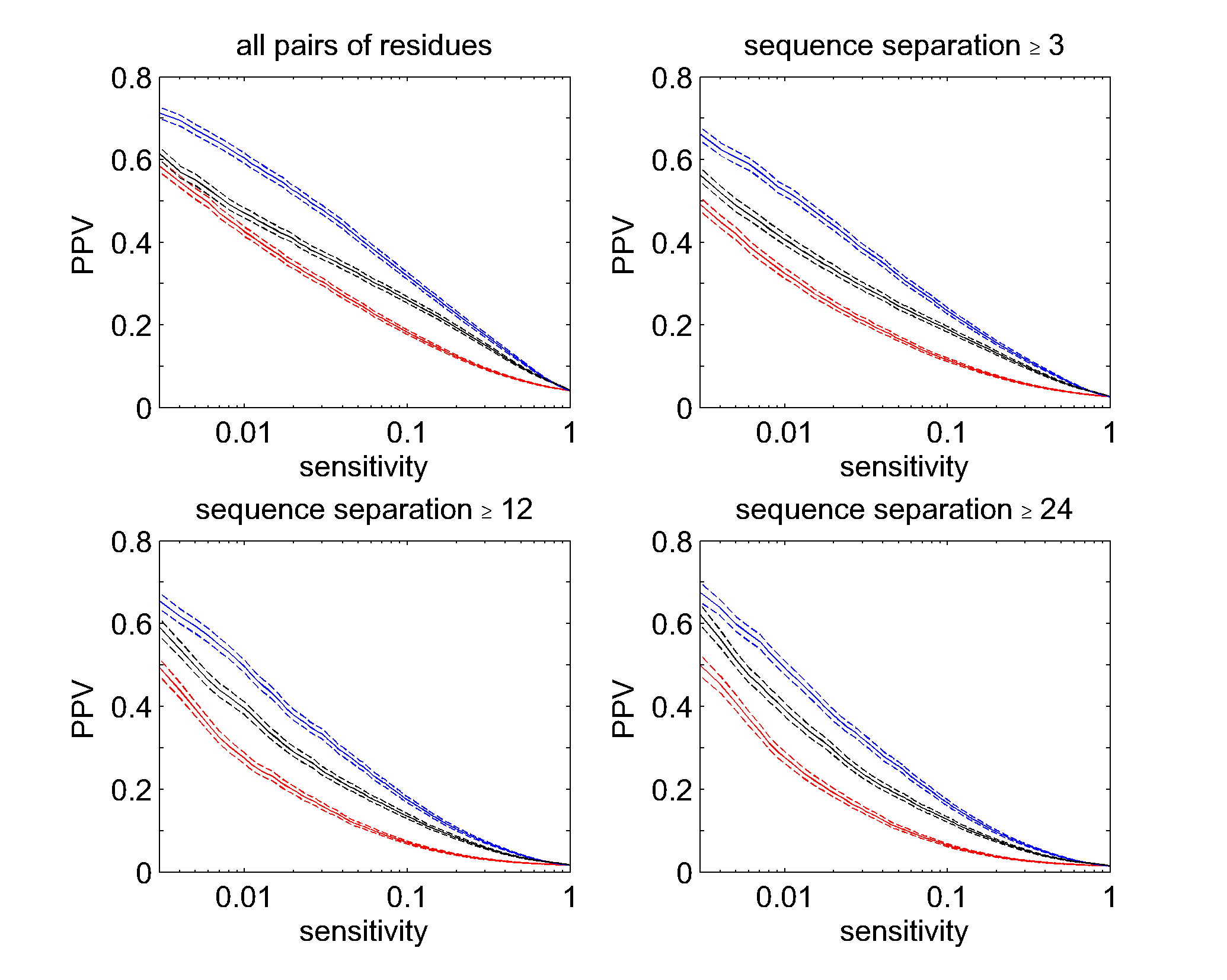

Supplement: Figure S8 — Accuracy of contact predictions for alignments of length 201 to 300. In blue, we show the performance of the phylogenetically-corrected posterior probabilities, in black the performance of the predictions based on the average-product corrected (APC) mutual information, and in red the performance of the posterior probabilities without phylogenetic correction. Curves were calculated as described in the main text. (0.33 MB TIF) [file pcbi.1000633.s008.tif]

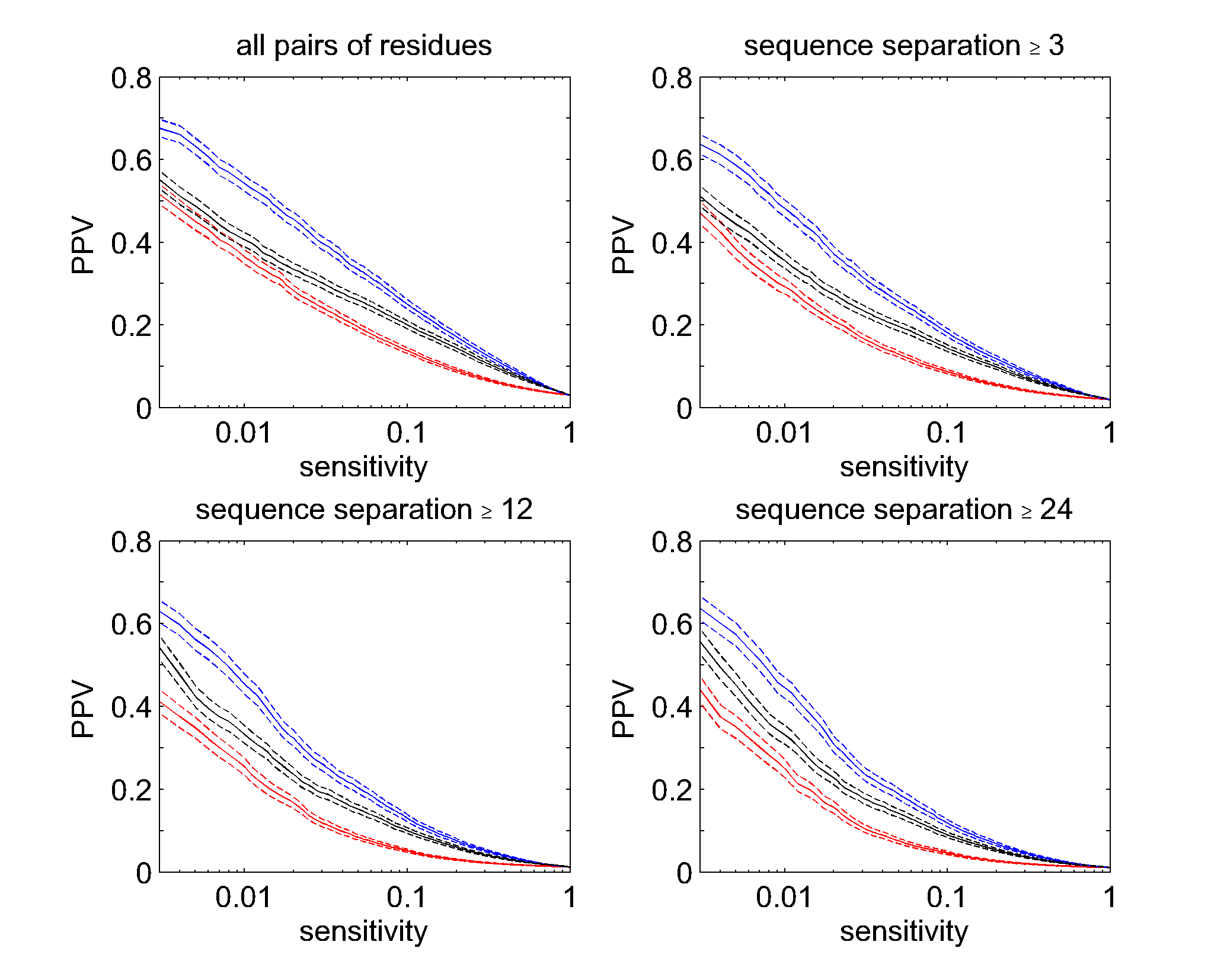

Supplement: Figure S9 — Accuracy of contact predictions for alignments of length 301 to 400. In blue, we show the performance of the phylogenetically-corrected posterior probabilities, in black the performance of the predictions based on the average-product corrected (APC) mutual information, and in red the performance of the posterior probabilities without phylogenetic correction. Curves were calculated as described in the main text. (0.33 MB TIF) [file pcbi.1000633.s009.tif]

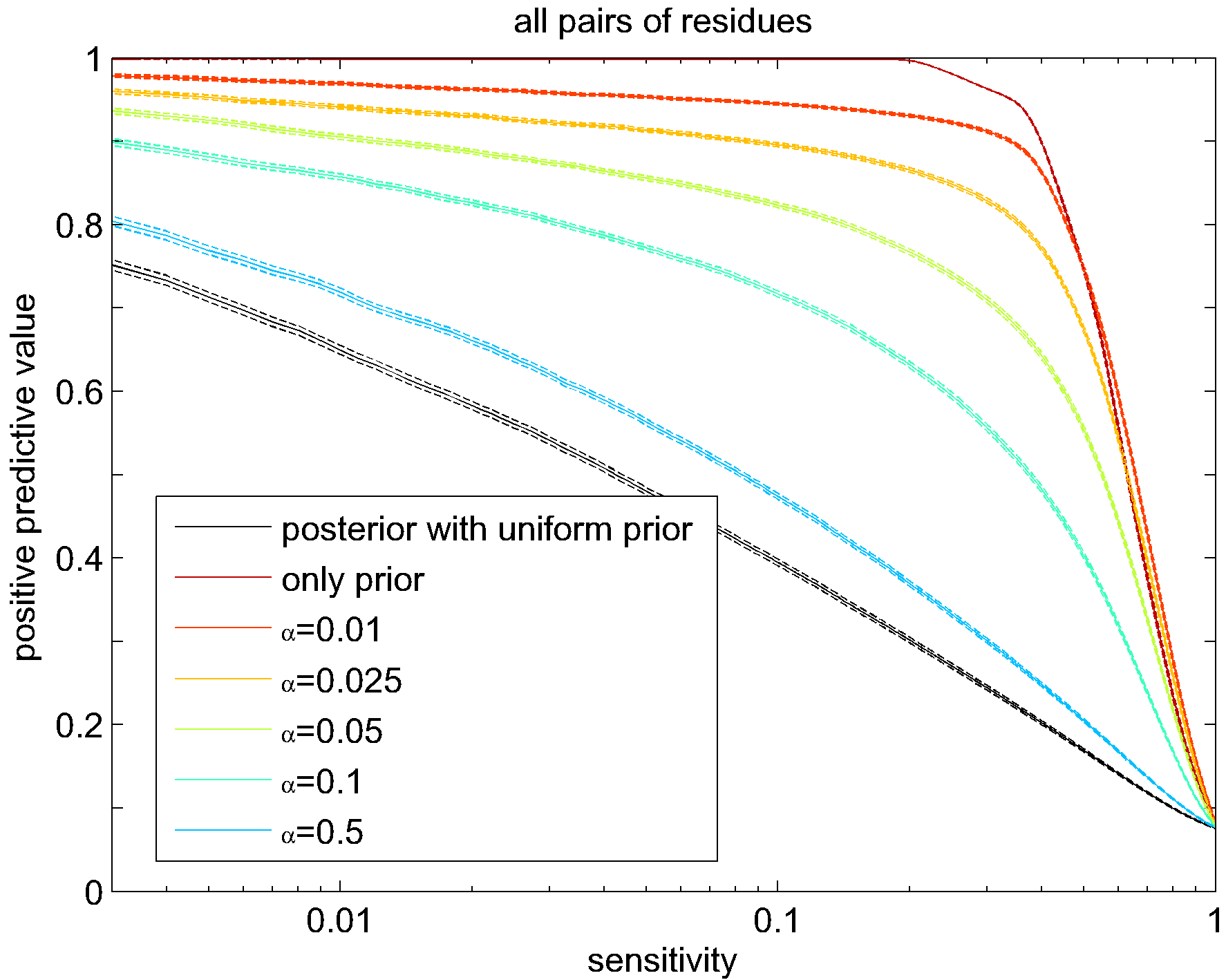

Supplement: Figure S10 — Accuracy of contact predictions including the informative prior for different values of the weighting parameter α, including the limit of using only the informative prior (α = 0). The positive predictive value (vertical axis) is shown as a function of sensitivity (horizontal axis). Different colors correspond to different values of α (see legend) and dashed lines show mean plus and minus one standard error. For comparison, we also show the performance of the posterior when using no prior information (black). Note that the horizontal axis is shown on a logarithmic scale. (0.37 MB TIF) [file pcbi.1000633.s010.tif]

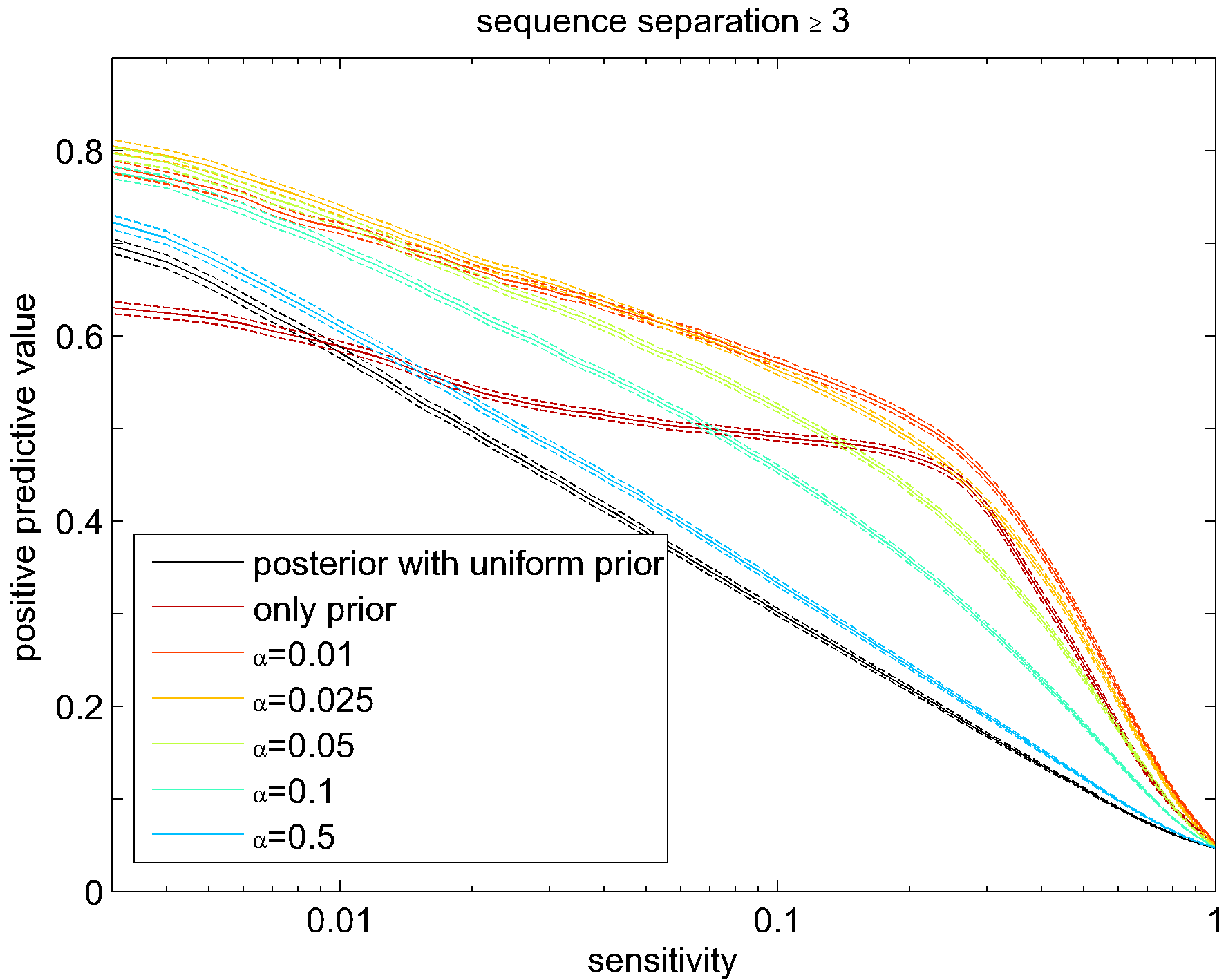

Supplement: Figure S11 — Accuracy of contact predictions including the informative prior for different values of the weighting parameter α, including the limit of using only the informative prior (α = 0), when considering only pairs that are at least d = 3 apart in primary sequence. The positive predictive value (vertical axis) is shown as a function of sensitivity (horizontal axis). Different colors correspond to different values of α (see legend) and dashed lines show mean plus and minus one standard error. For comparison, we also show the performance of the posterior when using no prior information (black). Note that the horizontal axis is shown on a logarithmic scale. (0.36 MB TIF) [file pcbi.1000633.s011.tif]

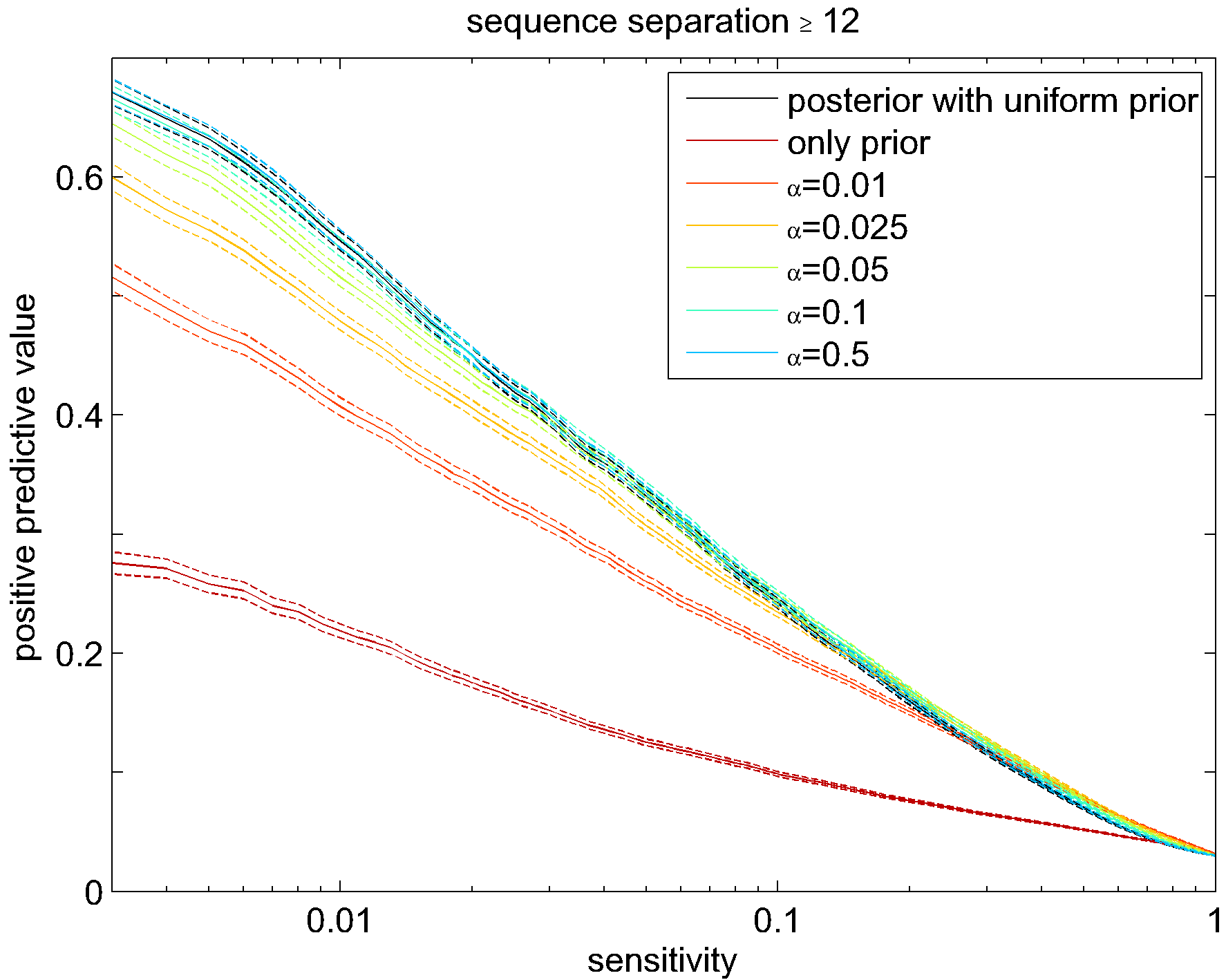

Supplement: Figure S12 — Accuracy of contact predictions including the informative prior for different values of the weighting parameter α, including the limit of using only the informative prior (α = 0), when considering only pairs that are at least d = 12 apart in primary sequence. The positive predictive value (vertical axis) is shown as a function of sensitivity (horizontal axis). Different colors correspond to different values of α (see legend) and dashed lines show mean plus and minus one standard error. For comparison, we also show the performance of the posterior when using no prior information (black). Note that the horizontal axis is shown on a logarithmic scale. (0.33 MB TIF) [file pcbi.1000633.s012.tif]
